# Oxygen adsorption on (100) surfaces in Fe–Cr alloys

**DOI:** 10.1038/s41598-021-85243-0

**Published:** 2021-03-15

**Authors:** Matti Ropo, Marko Punkkinen, Pekko Kuopanportti, Muhammad Yasir, Sari Granroth, Antti Kuronen, Kalevi Kokko

**Affiliations:** 1 Department of Physics and Astronomy, University of Turku, 20014 Turku, Finland; 2 Faculty of Engineering and Natural Sciences, Tampere University, 33014 Tampere, Finland; 3 Department of Physics, University of Helsinki, P.O. Box 43, 00014 Helsinki, Finland

**Keywords:** Electronic properties and materials, Electronic properties and materials, Chemical physics, Surfaces, interfaces and thin films

## Abstract

The adsorption of oxygen on bcc Fe–Cr(100) surfaces with two different alloy concentrations is studied using ab initio density functional calculations. Atomic-scale analysis of oxygen–surface interactions is indispensable for obtaining a comprehensive understanding of macroscopic surface oxidation processes. Up to two chromium atoms are inserted into the first two surface layers. Atomic geometries, energies and electronic properties are investigated. A hollow site is found to be the preferred adsorption site over bridge and on-top sites. Chromium atoms in the surface and subsurface layers are found to significantly affect the adsorption properties of neighbouring iron atoms. Seventy-one different adsorption geometries are studied, and the corresponding adsorption energies are calculated. Estimates for the main diffusion barriers from the hollow adsorption site are given. Whether the change in the oxygen affinity of iron atoms can be related to the chromium-induced charge transfer between the surface atoms is discussed. The possibility to utilize the presented theoretical results in related experimental research and in developing semiclassical potentials for simulating the oxidation of Fe–Cr alloys is addressed.

## Introduction

Iron–chromium alloys form the basis for the wide variety of transition metal alloys known as stainless steels. The most remarkable and distinct property of the stainless steels is their corrosion-resistant surface^1^. The corrosion resistivity is due to the protective, self-healing oxide layer, which has a complex structure containing $\hbox {Cr}_2 \hbox {O}_3$, $\hbox {Fe}_2 \hbox {O}_3$ and $\hbox {Fe}_3 \hbox {O}_4$ oxides^2–4^. In ferritic steels the corrosion rate drops dramatically when their chromium concentration increases to 9–10 at%^5^, and the steels become regarded as stainless. The onset of the decrease of the corrosion rate correlates with^6,7^ anomalous surface segregation of Cr that originates from the complex magnetic interactions between bulk and surface atoms^8,9^.

Due to its considerable economic importance, there has been a lot of interest in the oxidation of Fe–Cr alloys in scientific literature^10–13^. Yet the atomic-level understanding of the initial stages of oxidation of Fe–Cr surfaces, and how the oxide grows, is scarce. Investigations of the initial oxidation, especially computational works, have focused on cases of pure Fe and Cr. Yuan et al.^14^ performed calculations based on the density functional theory (DFT) with the generalized-gradient approximation (GGA) to investigate the effect of segregating alloying elements on the oxygen adsorption on Fe(100) surfaces. The effects of nine different 3d transition metals were investigated, and oxygen was found to be attracted to those alloying elements that have a lower atomic number than Fe. Błoński et al.^15^ investigated electronic and structural properties of oxygen adsorption on Fe(100) and Fe(110) surfaces. A twofold bridge site for (110) and a hollow site for (100) were found to be preferred. The effect of the oxygen coverage on electronic, magnetic and structural properties were investigated by Błoński et al.^16^, Tan et al.^17^ and Ossowski and Kiejna^18^ for Fe(100) and/or Fe(110) surfaces.

There are few experimental works on the initial or low-pressure oxygen adsorption for Fe or Fe–Cr alloys. Already in 1976 Leygraf and Hultquist^10^ investigated the initial oxidation of (110) and (100) surfaces in Fe and Fe–Cr using Auger electron spectroscopy (AES) and low-energy electron diffraction (LEED). They found that different oxides form on the (100) and (110) surfaces. On the (100) surface mixed Fe and Cr oxides are formed, whereas on the (110) surface only $\hbox {Cr}_2 \hbox {O}_3$, is formed preventing further oxidation. Using LEED, AES, electron-energy-loss spectroscopy (EELS), secondary-electron emission spectroscopy (SES) and work-function-change measurements, Sakisaka et al.^19^ found that the interaction of oxygen with the Fe(100) surface at 300 K consists of three stages: (i) dissociative chemisorption of oxygen at the hollow or bridge site, (ii) oxygen incorporation into the selvedge of the material, and (iii) formation of $\gamma$-Fe_2_O_3_. The magnetic properties of the initial oxygen adsorption for the (110) surface of Fe were investigated by Busch and Winter^20^ and by Getzlaff et al.^21^. Busch et al. focused on molecular oxygen on the Fe surface, whereas Getzlaff et al. focused on the atomic oxygen on the Fe surface. Initial oxidation of Fe–Cr has also been studied by medium-energy ion scattering (MEIS), Mössbauer and X-ray photoelectron spectroscopy (XPS)^2,22,23^.

For the initial oxidation of a Cr surface, only two computational investigations were found. Han and Liu^24^ have used a five-parameter Morse potential to study oxygen adsorption on the (100), (110), (111) and (211) surfaces of Cr. For the (100) surface a hollow site is preferred, whereas for the rest a quasi-threefold site is preferred. Zimmermann and Ciacchi^25^ have investigated initial oxidation and oxide formation for the Cr(110) surface using molecular dynamics simulations and static structural DFT calculations. They found that oxygen forms a perfect ad-layer before the actual formation of Cr oxides on the surface. More have been done experimentally for Cr surfaces: Müller and Oechsner^26^ investigated the initial oxidation of a Cr(110) surface and presented three different stages of oxidation. Peruchetti et al.^27^, Shinn and Madey^28^ and Baca et al.^29^ have investigated chemisorption of oxygen on Cr(100) and Cr(110) surfaces.

To our knowledge, there are only two computational studies that investigate oxygen adsorption on the Fe surface in the presence of Cr atoms: one by Han et al.^30^ and another by Yuan et al.^14^ In both studies the effect of alloying elements on the adsorption is investigated in the dilute limit with a single Cr atom in the surface. Han et al.^30^ investigated ten alloying elements in the $\gamma$-Fe(111) surface. They found that Cr has the strongest binding energy to oxygen and to water of investigated alloys. Yuan et al.^14^ studied the $\alpha$-Fe(100) surface and nine different alloying atoms in the surface. The hollow site was found to be preferred, followed by the bridge site and then the on-top site. The subsurface positions for oxygen were the least preferred positions. In both studies the alloying elements were placed only at one position in the surface.

This paper examines the adsorption of atomic oxygen to (100) surfaces of bcc Fe–Cr alloys with ab initio DFT calculations. We study the preferred adsorption sites, adsorption energies and how these are affected by the presence of Cr in the surface. We consider the effect of different surface Cr positions up to two Cr atoms in the surface. We also address the effect of the bulk composition of the Fe–Cr alloy on the adsorption. Since Fe–Cr alloys are also interesting in terms of magnetism, we further present a summary of the magnetic properties of the investigated surfaces.

Accurate and detailed atomic-scale data of the energetics and geometry of the adsorption processes of oxygen on Fe–Cr surfaces is essential not only for modeling the surface oxidation, but also for developing well-performing multi-targeted semiclassical potentials. Such potential models are essential for large-scale simulation methods that facilitate the efficient design of more sustainable iron alloys than has been achieved with trial and error.

## Methods

All ab initio density functional calculations are performed using GPAW^31,32^ (version 0.11) and the Atomic Simulation Environment (ASE)^33^ (version 3.9). The valence-core interaction is modeled with the projected augmented wave potentials (GPAW/PAW version 0.8), and a real-space grid with a 0.2-Å grid spacing is used to present the wavefunctions. A $3\times 3\times 1$ Monkhorst–Pack grid is used for the *k* points. A generalized-gradient-level approximation in the form of the Perdew–Burke–Ernzerhof^34^ functional is used for the exchange-correlation interaction. The calculations are done using a slab construction where the surface is modeled by a metal-vacuum film that is infinite in two dimensions and periodically repeates the metal-vacuum structure in the direction perpendicular to the film surface. The metal and vacuum parts should be thick enough to give converged results for the quantities to be calculated. Several useful convergence tests have been published. For instance, Yu et al.^35^ found that the computational accuracy of the surface energy of Fe(100) is 0.03% at a vacuum thickness of 8 Å. Moreover, we use a real-space grid technique in which net charges or dipoles present neither conceptual nor computational difficulties^36^.

The surfaces are modeled with five-atomic-layers-thick slabs with nine atoms in each layer. A 12-Å vacuum separates the surfaces. Simulating a dilute Fe–Cr alloy with a 45-atom unit cell, one or two Cr atoms are placed in the two topmost atomic layers, depending on whether adsorption with one or two Cr atoms is studied. To simulate the 9 at% Fe–Cr alloy, two of the Cr atoms are placed in the two bottommost atomic layers (maximally far from each other). Then additional one or two Cr atoms are placed in the two topmost atomic layers, as in the dilute Fe–Cr alloy case. In every calculation the atoms in the two bottommost layers (opposite to the adsorbed oxygen atom) are fixed to their bulk positions, and the rest of the atoms are allowed to relax using the FIRE^37^ algorithm with a relaxation criteria of 0.05 eV/Å. The theoretical lattice constants of 2.846 Å and 2.872 Å for pure Fe and Fe_0.91_Cr_0.09_ alloy are used. Atomic charges are calculated using the Bader method implemented in GPAW.

The surface energies are estimated using the formula (due to the asymmetric slab geometry only one of the surfaces is relaxed)

1 $$\begin{aligned} \gamma _\text {surface} = \frac{E_\text {slab}-n\,E_\text {bulk}}{A}-\gamma _\text {unrelaxed}, \end{aligned}$$

where $\gamma _\text {surface}$, $E_\text {slab}$, $E_\text {bulk}$, *n*, *A* and $\gamma _\text {unrelaxed}$ are the surface energy of the relaxed surface, the energy of the relaxed slab, the energy per atom for the bulk, the number of atoms in the slab, the area of the surface and the surface energy of the unrelaxed surface, respectively. The unrelaxed surface energy is calculated with the commonly used method of Ref.^38^.

The adsorption energies for an oxygen atom are calculated with the formula

2 $$\begin{aligned} E_\text {ad}=E^\text {slab+O}-E^\text {slab}-E^{\text {O}_2}/2, \end{aligned}$$

where $E^{\text{slab+O}}$, $E^{\text{slab}}$ and $E^{\text {O}_2}$ are the total energies of a slab with an adsorbed O atom, a slab without any oxygen and an oxygen molecule, respectively. A negative adsorption energy means adsorbate binding.

## Results

We investigate the oxygen adsorption on (100) surfaces of bcc Fe–Cr alloys. Calculations are performed with two different lattice constants: one set of calculations with a pure Fe lattice constant to simulate dilute-limit compositions and another set with a lattice constant corresponding to the Fe_0.91_Cr_0.09_ composition. The compositions are selected to present two distinct regions of corrosion resistance: in the dilute limit the corrosion rate is high, whereas at the Fe_0.91_Cr_0.09_ composition the corrosion rate is already reduced drastically^5^. The actual Cr concentrations of the dilute alloys with one or two Cr atoms in the surface are 2 at% or 4 at%, respectively, whereas the actual Cr concentration in the Fe_0.91_Cr_0.09_ case is either 7 at% or 9 at% depending on whether oxygen adsorption with one or two Cr atoms is studied.

**Figure 1:**
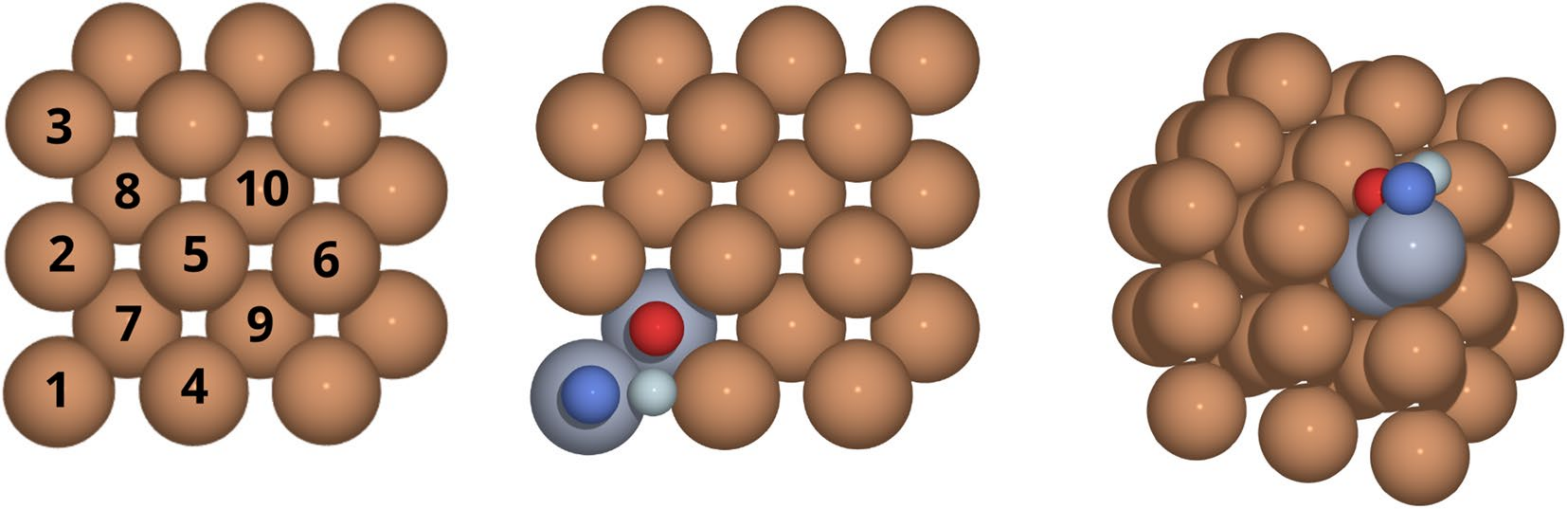
Left: Schematic illustration of the numbering of the Fe atoms in the two topmost atomic layers. The surface-layer atoms are numbered from 1 to 6 and the subsurface-layer atoms from 7 to 10. The three oxygen adsorption sites considered are the on-top site (‘ot’) over atom 1, the hollow site (‘ho’) over atom 7 and the bridge site (‘br’) between atoms 1 and 4. Middle: Atomic positions within the unit cell of the dilute Fe–Cr alloy with two Cr atoms (blue–grey) at sites 1 and 7. The positions of the adsorbed oxygen atom at the on-top, hollow and bridge adsorption sites are illustrated by the smaller dark blue, red and light blue spheres, respectively. Right: Same as Middle but with the viewpoint shifted so that the vertical positions of the oxygen atoms can be perceived.

The obtained results also shed light on whether the change in the lattice constant due to the change in the alloy composition affects the interactions between Fe, Cr and O in the surface. The surface of the simulation cell is illustrated in Fig. 1. For both sets of calculations, up to two Cr atoms (to enable the study of Cr–Cr interactions) are placed in the first two surface layers. To simulate the bulk concentration of the Fe_0.91_Cr_0.09_ alloys, two extra Cr atoms are placed in the two bottommost (opposite to the adsorption surface) layers of the simulation cell. The effect of the two extra Cr atoms on the interaction in the surface is estimated to be less than 1 meV for the full simulation cell. Three different adsorption sites are considered: *on-top* (‘ot’, on top of atom 1), *bridge* (‘br’, between atoms 1 and 4) and *hollow* (‘ho’, on top of atom 7) sites. For the numbering of the sites, see Fig. 1. A number is assigned to those first- and second-layer atomic sites that are needed to construct all non-equivalent atomic configurations (with respect to translation, rotation and mirror symmetries) for oxygen adsorption at the on-top, bridge and hollow sites with one or two Cr atoms substituted for Fe atoms in the first or second atomic layers.

### Surface energy and relaxation: oxygen-free surface

First we consider oxygen-free surfaces. The obtained surface energies are presented in Table 1, along with two DFT reference values for pure Fe calculated using a GGA-level exchange-correlation potential and the VASP program^38,39^ or the FCD-LMTO method^40^. Our estimate is well in line with the previous VASP results.

In addition to the surface energies, Table 1 lists the relaxations $\Delta _{ij} = 100(d_{ij}-d)/d$ for the two topmost surface layers; here $d_{ij}$ and *d* are the interlayer distances between the layers *i* and *j* and in the bulk, respectively. Our results for the clean Fe surface with the pure-Fe lattice constant are somewhat smaller than the corresponding VASP results^15^. For both investigated lattice constants, the single Cr atom prefers the top surface layer over the subsurface layer. In the case of two Cr atoms in the surface, both of them prefer to lie in the top layer, namely, at sites 1 and 5 in Fig. 1 (or other symmetrically equivalent configurations). This result is in agreement with previous first-principles calculations^8,41^. A detailed list of the energies of all calculated atomic configurations is shown in Supplementary Information.

**Table 1:** Surface energies and relaxations of the first two surface layers of the investigated systems.

System	surface	$\gamma _\text {surf}$ ($\hbox {J m}^{-2}$)	$\Delta _{12} (\%)$	$\Delta _{23}$ (%)
Fe	Fe	2.492	$-\,2.51$	1.43
FeCr	Fe	2.422	$-\,4.12$	1.31
Ref.^15^	Fe		$-\,3.03$	2.14
Refs.^38,39^	Fe	2.50		
Ref.^40^	Fe	2.430		

The system label ‘Fe’ indicates that the lattice constant of pure Fe (dilute alloy) is used; ‘FeCr’ indicates the lattice constant of the Fe_0.91_Cr_0.09_ alloy. Here $\Delta _{12}$ is the percentage change in the distance between the surface layer and the first subsurface layer, relative to the layer distance in bulk, and $\Delta _{23}$ is the percentage change in the distance between the first and second subsurface layers.

### Oxygen adsorption: preferred sites and geometries

To study oxygen adsorption, both for pure Fe and for Fe_0.91_Cr_0.09_ alloy with all possible substitutional Cr configurations in the two topmost layers, we consider three adsorption sites: the on-top site over atom 1, the hollow site over atom 7 and the bridge site between atoms 1 and 4 (Fig. 1). Given that surface adsorption generally alters surface electrostatics, the following remark about these adsorption configurations is in order: If the metal film is asymmetric, it is possible that a spurious dipole interaction forms between the adjacent metal films. Oxygen adsorption on an Fe surface increases the surface dipole moment. Hugosson et al.^42^ showed that 0.25 monolayer oxygen increases the surface dipole moment by 0.035 eÅ and 1 ML of oxygen increases it by 0.087 eÅ [here one monolayer (1 ML) adsorption: Fe(100) − p($1\times 1$)O]. Therefore, from the surface-dipole point of view, our atomic slab with 0.11 ML oxygen is close to a symmetric slab, which renders the dipole correction less important^43,44^.

When it comes to oxygen adsorption, there are only a few differences between the two investigated alloys. For both alloys the fourfold hollow site is the preferred site (Fig. 3); the bridge site is the second most favourable and the on-top site the least favourable. The same order was reported for oxygen adsorption on Fe(100) surfaces by Yuan et al.^14^. For the adsorption geometries, the oxygen–metal distances for the two investigated alloys are the same within $\pm \,0.01$ Å. For the oxygen at the on-top position, the distance between the oxygen and the underlying metal atom (be it either Fe or Cr) is 1.64 Å.

**Figure 2:**
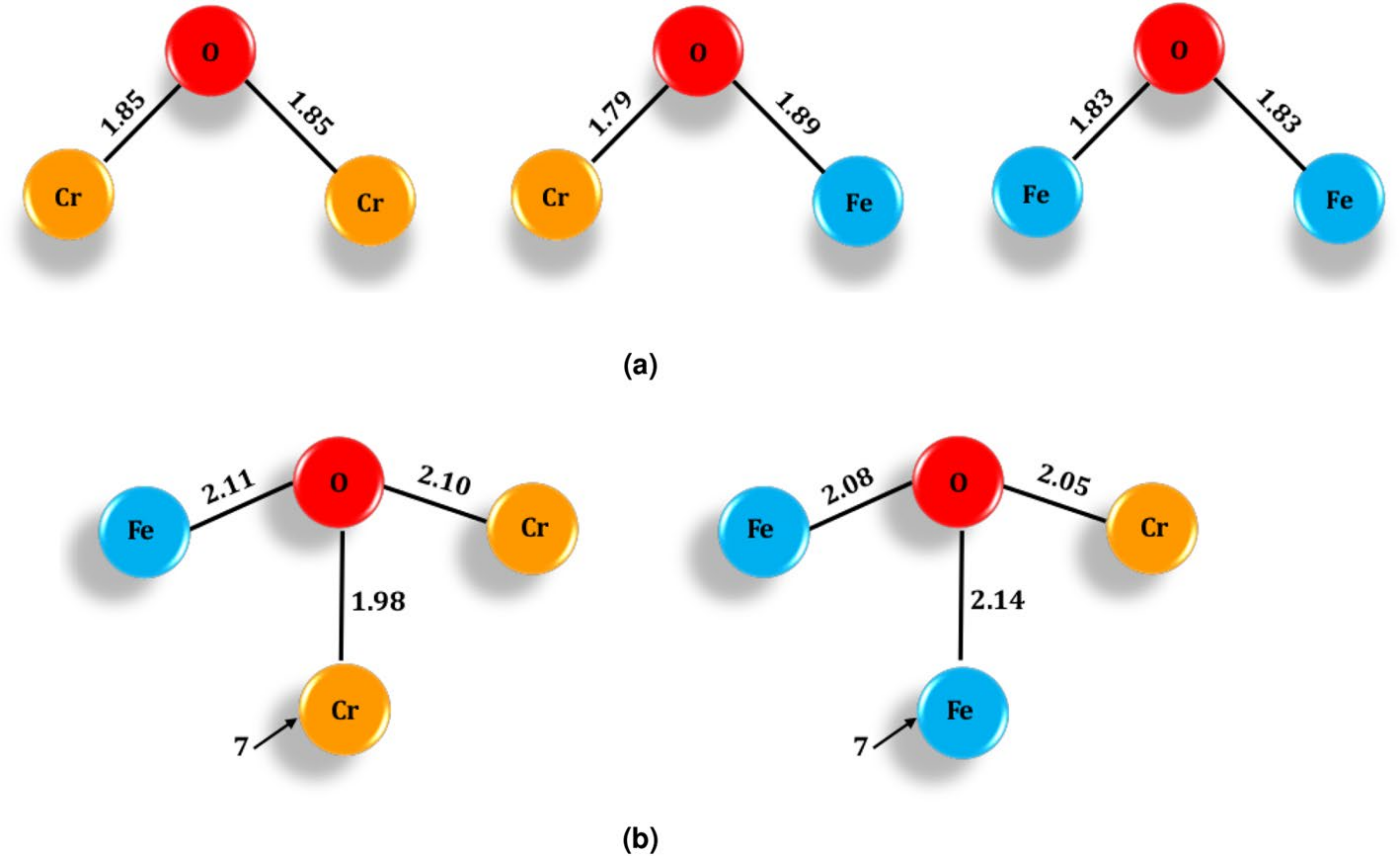
Schematic figure of the distances (in  Å) between an adsorbed oxygen atom and the nearest metal atoms. Upper panel: Oxygen atom adsorbed to the bridge site atoms, at atomic sites 1 and 4 (see Fig. 1 for the numbering of the sites). Lower panel: Oxygen atom at the hollow site, the Fe and Cr atoms on the side of the oxygen are atoms at sites 1 and 5.

When the oxygen is at the bridge position, the atomic distances depend on the type of the bridge dimer below the oxygen. The distances between the oxygen and the metal atoms are shown schematically in Fig. 2a. Note that the two oxygen–metal distances in the Cr–Fe bridge differ significantly (6%) from each other, the O–Cr bond being shorter; their average, however, is 1.84 Å, which is equal to the average bond distance of the Cr–Cr and Fe–Fe cases. The DFT calculations with the Perdew–Burke–Ernzerhof exchange-correlation functional for a pure Fe surface by Yuan et al.^14^ yield similar results: 1.63 Å for the on-top and 1.83 Å for the bridge position. The adsorption energy of an oxygen atom at a bridge site depends almost linearly on the type of the bridge atoms: For both alloy compositions, the adsorption energy for the Fe–Fe bridge is − 3.24 eV (Table 2). It decreases by about 0.3 eV for the Cr–Fe bridge and again by about 0.3 eV for the Cr–Cr bridge for both alloys. This gives approximately − 1.6 eV per O–Fe bond and − 1.9 eV per O-Cr bond.

For the hollow site the behaviour is more intricate. The distance to the first layer depends on which atom is underneath the oxygen atom (at site 7). Also, in the case of hollow adsorption, if another Cr atom is replaced by an Fe atom, the distance between the remaining Cr atom and the oxygen atom is shortened, just as in the bridge case. Although the individual distances from the hollow-site oxygen atom to the five nearest atoms depend on whether the atom below the oxygen is iron or chromium, the average distance to the five nearest atoms is essentially the same in both cases (differing by only 0.5%); this is again similar to the behaviour of the bridge dimer. The distances in the hollow case are illustrated in Fig. 2b. Yuan et al. reported the shortest bond length between Fe and O for the hollow site of a pure Fe surface to be 2.05 Å^23^.

### Adsorption energies

The obtained adsorption energies for zero, one and two Cr atoms in the surface are given for the dilute Fe–Cr alloy and Fe_0.91_Cr_0.09_ in Table 2.

**Figure 3:**
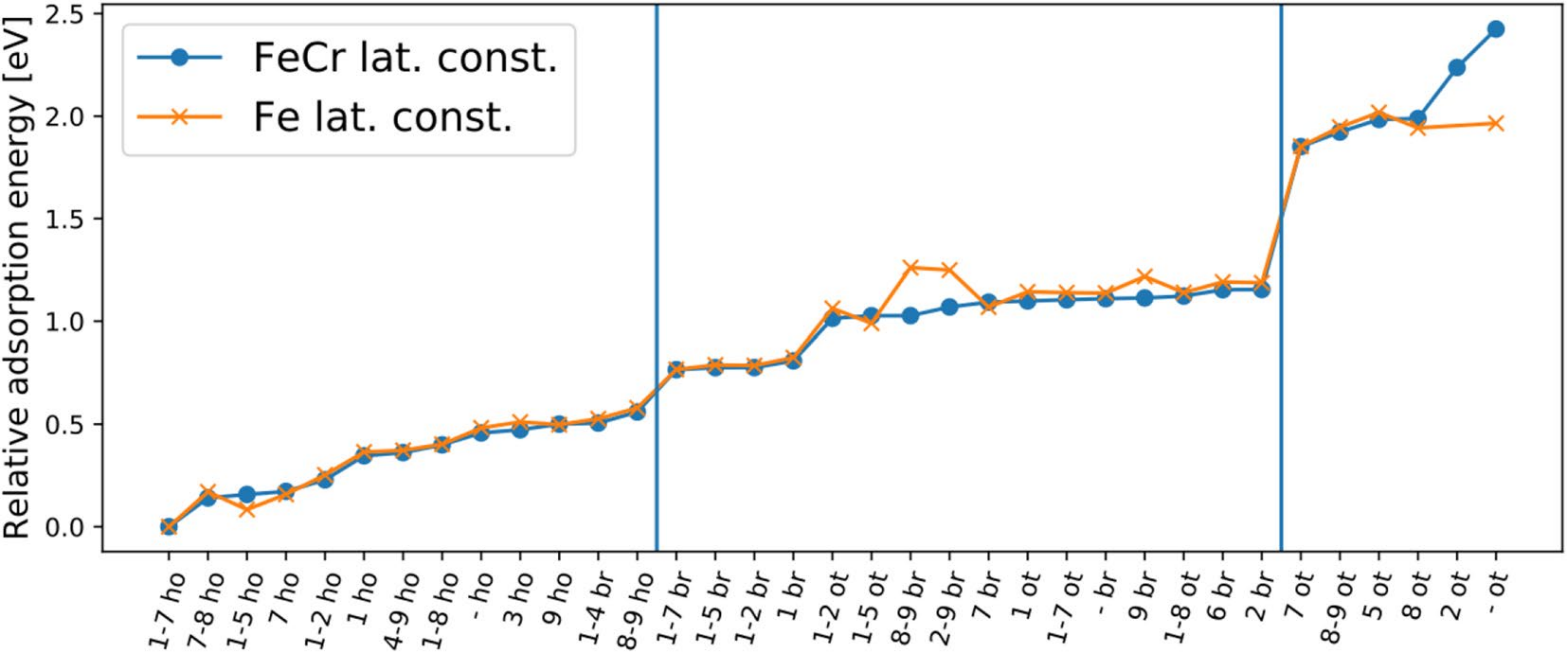
Relative adsorption energies of oxygen in the Fe and Fe_0.91_Cr_0.09_ systems (the energy of the ‘1–7 ho’ case is shifted to 0 eV for both systems, with $E_\text {ad}=-\,4.37$ eV and $-\,4.35$ eV for the Fe and Fe_0.91_Cr_0.09_ ‘1–7 ho’ cases, respectively). The difference between the two systems is minimal except for a few cases. The sites have the following labels: ‘br’ is a bridge site, ‘ot’ is an on-top site and ‘ho’ is a hollow site. The numbers in front indicate the position(s) and number of Cr atoms in the surface: one number indicates one Cr atom, and two numbers separated by a hyphen indicate two Cr atoms in the surface; a hyphen without any numbers indicates a pure Fe surface. The atomic sites are numbered as in Fig. 1. The left (right) vertical line separates all the hollow (bridge) adsorption cases to its left. From the point of view of energetic stability (see Supplementary Information), the most relevant configurations are those that have Cr only in the surface layer, i.e. at sites from 1 to 6.

**Table 2:** Adsorption energies (in  eV) of an oxygen atom calculated using the lattice constant of pure iron (‘Fe’) and the lattice constant of Fe_0.91_Cr_0.09_ (‘FeCr’).

Cr pos.	O pos.	$E_\text {ad}$ (eV)	
		Fe	FeCr
–	br	$-\,3.24$	$-\,3.24$
–	ho	$-\,3.89$	$-\,3.90$
–	ot	$-\,2.41$	$-\,1.93$
1	br	$-\,3.55$	$-\,3.54$
2	br	$-\,3.19$	$-\,3.20$
6	br	$-\,3.18$	$-\,3.20$
7	br	$-\,3.30$	$-\,3.26$
9	br	$-\,3.16$	$-\,3.24$
1	ho	$-\,4.01$	$-\,4.01$
3	ho	$-\,3.86$	$-\,3.88$
7	ho	$-\,4.22$	$-\,4.18$
9	ho	$-\,3.88$	$-\,3.85$
1	ot	$-\,3.23$	$-\,3.25$
2	ot		$-\,2.12$
5	ot	$-\,2.36$	$-\,2.37$
7	ot	$-\,2.52$	$-\,2.50$
8	ot	$-\,2.43$	$-\,2.36$
1–2	br	$-\,3.59$	$-\,3.58$
1–4	br	$-\,3.85$	$-\,3.85$
1–5	br	$-\,3.58$	$-\,3.58$
1–7	br	$-\,3.61$	$-\,3.59$
2–9	br	$-\,3.12$	$-\,3.28$
8–9	br	$-\,3.11$	$-\,3.21$
1–2	ho	$-\,4.12$	$-\,4.12$
1–5	ho	$-\,4.21$	$-\,4.20$
1–7	ho	$-\,4.37$	$-\,4.35$
1–8	ho	$-\,3.97$	$-\,3.95$
4–9	ho	$-\,4.00$	$-\,4.00$
7–8	ho	$-\,4.20$	$-\,4.21$
8–9	ho	$-\,3.79$	$-\,3.79$
1–2	ot	$-\,3.31$	$-\,3.34$
1–5	ot	$-\,3.30$	$-\,3.33$
1–7	ot	$-\,3.23$	$-\,3.25$
1–8	ot	$-\,3.23$	$-\,3.23$
4–8	ot	$-\,3.55$	$-\,3.54$
8–9	ot	$-\,2.43$	$-\,2.42$

For instance, the notation ‘1–2 br’ means that there are Cr atoms at sites 1 and 2 (see Fig. 1 for the site numbering) and that the oxygen atom is adsorbed at the bridge position. The sites have the following labels: ‘br’ is the bridge site, ‘ot’ is the on-top site, and ‘ho’ is the hollow site.

**Figure 4:**
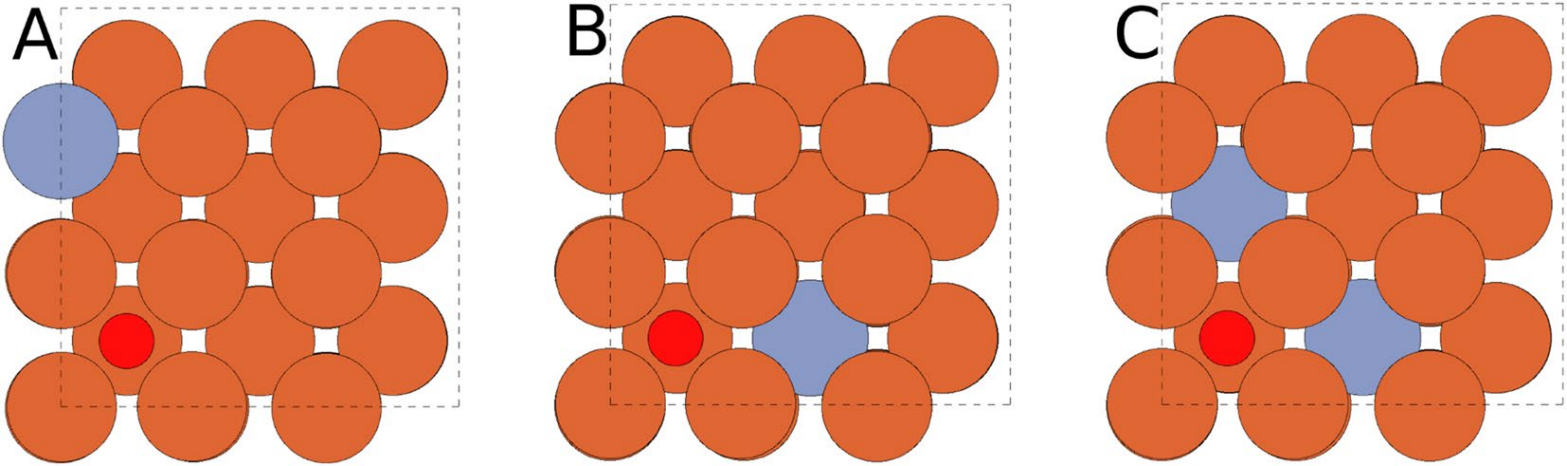
Illustrations of the three hollow-site configurations in which oxygen is more weakly bound than at the hollow site of a pure Fe surface. The positions of Cr atoms (indicated with blue-gray color) are (**A**) site 3, (**B**) site 9 and (**C**) sites 8 and 9. The oxygen position is indicated by the small red sphere.

As mentioned in the previous section, the strongest binding site is a hollow one. The configuration with Cr atoms at sites 1 and 4 is the strongest binding bridge case. In fact, its binding is stronger than that of a hollow site in a configuration where the Cr atoms are at sites 8 an 9. Sites 8 and 9 are next-nearest neighbours to site 7 directly below the hollow site, which suggests that the Cr effect on the oxygen adsorption is predominantly of a short-range nature (see Fig. 4C).

Interestingly, only three of the eleven investigated Cr-containing hollow-adsorption cases (‘3 ho’, ‘9 ho’, ‘8–9 ho’) are less binding than the pure-Fe-surface hollow site (‘- ho’) (Table 2 and Figs. 3, 4). In these three cases, there are no Cr atoms within the nearest-neighbour positions of the adsorption site. Placing Cr at site 3, site 9 or sites 8 and 9 raises the adsorption energy of oxygen by 0.016 eV, 0.029 eV or 0.097 eV, respectively. The effect of Cr on the adsorption of oxygen is therefore ambivalent, that is, Cr within the nearest-neighbour distance from the adsorption site enhances oxygen adsorption and at farther distances weakens the oxygen adsorption. Similar behaviour can also be observed for the bridge position in both investigated alloys and for the on-top position in the case of the pure-Fe lattice constant. For the Fe_0.91_Cr_0.09_ alloy with oxygen at the on-top position, the pure Fe surface has the weakest oxygen binding. For the dilute-limit alloy, the weakest binding occurs when the Cr atom is at site 5 (‘5 ot’ in Table 2; see also Figs. 1, 3). The overall difference in the adsorption energies between the dilute-limit Fe–Cr and Fe_0.91_Cr_0.09_ alloys is small. The mean difference is $\sum (E_\text {ad}(\text {Fe})-E_\text {ad}(\text{Fe}_{0.91}\text {Cr}_{0.09}))/N = -\,0.008$ eV, and the mean absolute difference is $\sum \left|(E_\text {ad}(\text {Fe})-E_\text {ad}(\text {Fe}_{0.91}\text {Cr}_{0.09}))\right|/N = 0.037$ eV; the sum is over identical surface configurations with $N = 35$.

The energetic stability of the considered Cr configurations can be assessed using the Maxwell–Boltzmann statistical distribution and the total energies of systems with different Cr configurations. The relative probability of configurations *i* and *j* with energies $E_i$ and $E_j$ at temperature *T* is $\exp {[(E_j-E_i)/(kT)]}$, where *k* is the Boltzmann constant. To avoid biased energies between systems with different numbers of substituted Cr atoms, we consider the systems with one and two substitutional Cr atoms in the surface region of the unit cell as separate sets in the probability calculations. Both concentration and temperature affect the occurrence probabilities of Cr configurations in iron alloys. In order to get a broader view of the Fe–Cr alloys, it is worth mentioning some of their general properties^6,8,41^. When the Cr concentration reaches about 10 at% in bulk, the probability of finding Cr in the surface starts to increase steeply above the bulk value. The occurrence probability of a second-layer Cr atom stays lower than that of a surface-layer Cr atom. Moreover, the occurrence probability of a Cr dimer decreases with decreasing distance between the Cr atoms. At higher temperatures, higher-energy Cr configurations become more probable. At a temperature of 300 K, the second surface layer contains virtually no chromium. The probabilities of the ‘1–5’ and ‘1–2’ configurations are, respectively, 96% and 4% in the dilute Fe–Cr alloy and 97% and 3% in the Fe_0.91_Cr_0.09_ alloy. At 1100 K, the probabilities of the ‘1–5’ and ‘1–2’ configurations are 68% and 29% in the dilute Fe–Cr alloy and 71% and 28% in the Fe_0.91_Cr_0.09_ alloy; the negligible 300 K probability of the ‘1–8’ configuration has increased to about 2% for the dilute Fe–Cr alloy and to 1% for the Fe_0.91_Cr_0.09_ alloy.

Introducing oxygen onto the surface changes the energetic stability of the Cr configurations; the magnitudes of the changes range from a few percents at room temperature up to tens of percents at high temperatures. At 1100 K, the probabilities of the ‘1–5’ and ‘1–2’ configurations are, respectively, 81% and 14% in the dilute Fe–Cr alloy and 81% and 15% in the Fe_0.91_Cr_0.09_ alloy. The negligible 300 K probability of the ‘1–7’ configuration has increased to approximately 4% for the dilute Fe–Cr alloy and 2% for the Fe_0.91_Cr_0.09_ alloy. At temperatures where metal atoms become mobile, the adsorbing oxygen could change the atomic configuration of the Fe–Cr surface. Temperature, Cr concentration and oxidation can thus significantly alter the stability of the Cr configurations. The stabilities of the Cr configurations in Table 2 at temperatures of 300 K, 700 K, 1100 K and 1500 K are given in Supplementary Information.

In the case of the Fe surface, our results can be compared with previous investigations of the Fe surface. Cao^45^ reports the DFT-GGA values $-\,7.577$ eV, $-\,6.632$ eV and $-\,5.585$ eV for the oxygen adsorption energies for the hollow, bridge, and on-top adsorption sites on a Fe(100) surface, respectively. In Cao’s results, the reference level includes the energy of a free oxygen atom, in contrast to half the energy of a free oxygen molecule in our case. Therefore, to compare Cao’s results with our adsorption energies (Table 2), we must add half the binding energy of an oxygen molecule ($\frac{1}{2}\times 6.07$ eV^46^) to Cao’s results, yielding $-\,4.542$ eV, $-\,3.597$ eV, $-\,2.550$ eV. These differ from our results by $-\,0.7$ eV, $-\,0.4$ eV and $-0.2$ eV, respectively. However, also the oxygen coverage differs: in our calculations it is 0.11 monolayers (ML), whereas in Cao’s work it is 0.25 ML. Previous investigations have shown that the adsorption energy of oxygen decreases with increasing oxygen coverage. Błoński et al.^15^ report the DFT-GGA adsorption energies for the oxygen adsorption at hollow site on Fe(100) to be $-\,3.41$ eV, $-\,3.26$ eV and $-\,3.09$ eV for the coverages 0.25, 0.5 and 1.0 MLs, respectively. The trend of oxygen binding becoming weaker with increasing adsorbate coverage is also observed for other metal surfaces, such as Pd(111)^47^, Pt(111)^48^ and Au(111)^49^. Using UV and X-ray photoelectron spectroscopy, Maschhoff and Armstrong^50^ investigated the initial oxidation of polycrystalline Fe surface from atomic adsorption to $10^{5}$ Langmuir (L) exposure in ultra-high vacuum and up to oxidation in atmospheric conditions. They found that the initial oxide is FeO. After 10 L oxygen adsorption, $\hbox {Fe}_3 \hbox {O}_4$ starts to form.

The obtained adsorption energies are useful data, for instance, in Monte Carlo simulations of the growth of the oxide scale on pristine Fe and Fe–Cr (100) surfaces. These simulations could provide useful information about the differences in the oxidation process between corrosion-resistant and corrosion-susceptible surfaces. Having a comprehensive atomic picture of the oxidation processes of Fe and Fe–Cr surfaces would be very beneficial for modern alloy design.

### Electric charges

Before discussing electric charges of the atoms, we would like to point out that the charge of an atom in a solid is not an observable but rather relies on a model used to partition the total charge density of the solid^51^. Nevertheless, relative changes in atomic charges, calculated using the same method for all systems, can give relevant physical and chemical information about the atomic processes. The electric charges of the atoms in the investigated systems are calculated with the Bader program^52,53^. The Bader method has been benchmarked and tested in several works^53–59^. For instance, Bader charges have been tested for Na metal using two different integration methods, the near-grid method and the weight method. For $60^3$ grid points, the near-grid method underestimates the Bader charge by 0.01 *e* (*e* is the absolute value of the charge of an electron), while the weight method underestimates it by 0.005 *e*.

Here again there are no significant differences in the charges between the two investigated systems (dilute and Fe_0.91_Cr_0.09_ alloys). The maximum charge difference between the two systems for the same configuration is $\pm \,0.07$ *e*. For the clean Fe surface, the average charge of the Fe atom is 0.09 *e* in the surface layer and - 0.10 *e* in the subsurface layer. (Here a charge is the difference between the Bader charge of an atom in the material and the electric charge of a free atom, i.e. the positive value indicates electron deficiency). The charge of a single Cr atom in an oxygen-free Fe surface layer is 0.38 *e*. Yuan et al.^14^ reported a charge of about 0.5 *e* for Cr in the Fe surface layer. Our result for a single Cr atom in the subsurface layer is 0.36 *e*. In the case of two Cr atoms in the first two surface layers, we obtain the average charges of 0.41 *e* and 0.31 *e* for Cr in the surface and subsurface layers, respectively.

When there is an oxygen atom at the on-top position (above site 1), it has an average charge of − 0.80 *e*, and the averages for the metal atoms directly below the oxygen are 0.74 *e* for Cr and 0.33 *e* for Fe. When the oxygen is at the bridge site, it has a charge of − 0.95 *e*, and the averages for the nearest metal atoms (sites 1 and 4) are 0.72 *e* for Cr and 0.40 *e* for Fe. In the case of oxygen at the hollow position, its average charge is − 1.15 *e*, and the averages for the nearest metal atoms are as follows: first-layer Cr 0.60 *e*, first-layer Fe 0.27 *e*, second-layer Cr 0.27 *e* and second-layer Fe 0.08 *e*.

### Electronic properties

To understand the intricate interactions between iron, chromium and oxygen, we have investigated the highest occupied (HO) states, the lowest unoccupied (LU) states and the density of states (DOS). The analysis reveals that the HO states are mainly localized at the Fe atoms whereas the Cr atom (or atoms) contributes strongly to the LU states whenever it is present. Similar DFT-GGA results were reported by Hu et al.^60^ for a single Cr atom in the (110) surface. These conclusions are also supported by our analysis of the local density of states (LDOS) using projections to the atomic basis. The Fe atoms have large contributions just below the Fermi level, whereas the Cr atoms have large contributions just above the Fermi level. Previous DFT calculations with the local density approximation by Papanikolaou et al.^61^ similarly revealed a large Cr contribution above the Fermi level in the LDOS of the Cr-containing Fe(100) surface.

**Figure 5:**
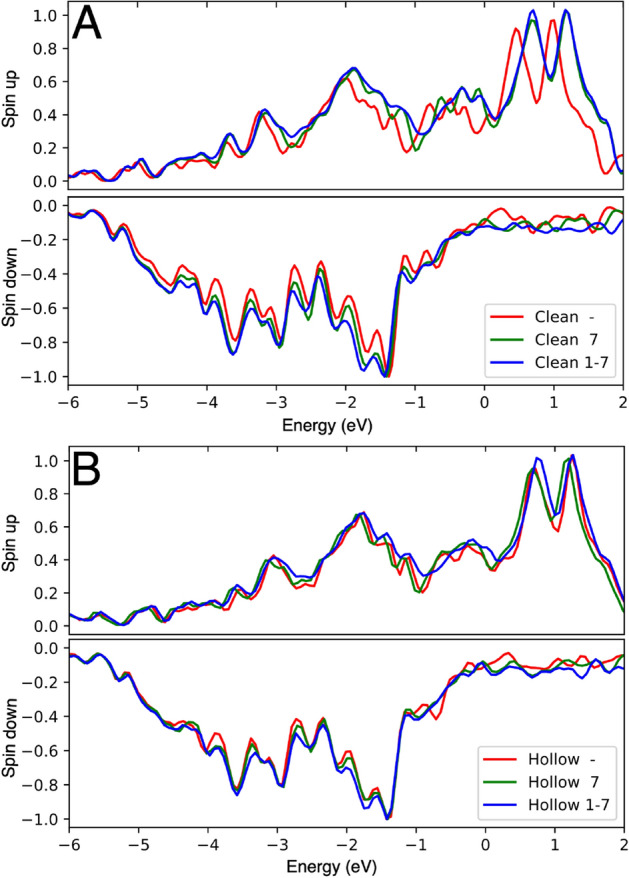
DOSes (positive values for up and negative values for down spin channels) for (**A**) an oxygen-free surface with zero (red), one (green) or two (blue) Cr atoms corresponding to the strongest binding configuration; (**B**) as (**A**) but with an oxygen atom at the hollow site. The horizontal axis is energy in  eV relative to the Fermi energy, and the vertical axis is the DOS in arbitrary units. The numbers in the legends indicate the position(s) of the Cr atom(s) (see Fig. 1 for the numbering scheme). The lone hyphen indicates the absence of Cr atoms in the surface.

Figure 5 shows the DOSes for pure and Cr-containing Fe surfaces in both oxygen-free (Fig. 5A) and oxygen-containing (Fig. 5B) cases, with the configuration corresponding to the strongest binding case for the hollow site. For the oxygen-free surfaces (Fig. 5A) there are clear differences between DOSes of pure and Cr-containing Fe surfaces (most clearly seen in the double peak at the top of the up d band). Chromium atoms increase the difference (spin splitting) between the up and down DOSes (by about 0.2 eV, measured for the DOS peaks at the top of the up and down d bands). An oxygen atom on the surface also increases the spin splitting (0.2 eV), as observed by comparing Fig. 5A,B. However, after adding oxygen on the surface the effect of chromium atoms on the spin splitting is considerably reduced (Fig. 5B).

**Figure 6:**
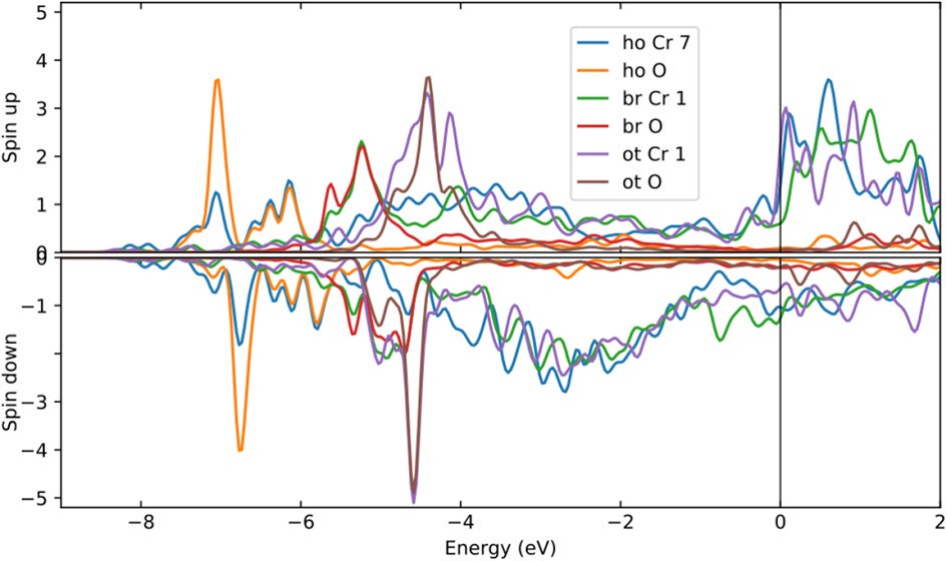
Comparison of the local densities of states of oxygen and chromium atoms calculated with a projection to an atomic basis located at the atomic sites. The labels ‘ho’, ‘br’ and ‘ot’ indicate whether the oxygen is at a hollow, bridge or on-top site. The second label tells the type of atom under consideration (Cr or O), and the number indicates the Cr position (see Fig. 1 for the site numbering). The horizontal axis is energy in  eV relative to the Fermi energy, and the vertical axis is the DOS (both up and down spin channels) in arbitrary units.

As mentioned above, LDOSes were also analysed for selected atomic sites to shed light on the behaviour of different atoms in different configurations. The LDOSes for the adsorbed oxygen atom, as well as for chromium atoms near the oxygen atom, are shown in Fig. 6 for oxygen at on-top, bridge and hollow sites. The states of the adsorbed oxygen are much lower (around 7 eV below the Fermi level) for the hollow site than they are for the bridge and on-top sites (around 5 eV and 4 eV below the Fermi level, respectively). There is also a strong overlap between some of the chromium and oxygen states, just below − 6 eV for the hollow site, below − 5 eV for the bridge site and below − 4 eV for the on-top site. The Fe DOS shows similar behaviour, although its overlap with oxygen is not as strong as that of chromium. As Fig. 6 shows, the band energy ($\int _0^{E_F} E[\text {DOS}_{\uparrow }(E)-\text {DOS}_{\downarrow }(E)]\text{d}E$) of oxygen decreases with the adsorption sites in the order ot–br–ho, in agreement with the adsorption energies of these sites. The strong overlap between oxygen and chromium states at low energies suggests stronger bonding of oxygen to chromium than to iron.

### Magnetic properties

Here we mainly focus on the magnetic-moment data of the dilute Fe–Cr alloy (data for Fe_0.91_Cr_0.09_ is given in parentheses). The atomic magnetic moments for all calculated Cr configurations are presented in Supplementary Information. The moments of Fe atoms in Cr-free surfaces are 2.968 $\mu _\text {B}$ (2.963 $\mu _\text {B}$) for an atom in the first layer and 2.340 $\mu _\text {B}$ (2.398 $\mu _\text {B}$) for an atom in the second layer. For comparison, the magnetic moment of bulk Fe is 2.186 $\mu _\text {B}$ per atom. The substitution of one Fe atom by a Cr atom reduces the moments of the nearby Fe atoms, on average, to 2.884 $\mu _\text {B}$ (2.865 $\mu _\text {B}$) in the surface layer and to 2.228 $\mu _\text {B}$ (2.248 $\mu _\text {B}$) in the second layer. Two Cr atoms, placed in the first or second (or both) atomic layer(s), reduce the moment of an Fe atom in the surface layer to 2.752 $\mu _\text {B}$ (2.769 $\mu _\text {B}$ ) and in the second layer to 2.198 $\mu _\text {B}$ (2.219 $\mu _\text {B}$). In general, there are only minor differences in magnetic moments between the dilute Fe–Cr alloy and Fe_0.91_Cr_0.09_ alloy.

The magnetic moment of a single Cr in the first layer is $-3.133$ $\mu _\text {B}$ ($-3.149$ $\mu _\text {B}$) and $-1.984$ $\mu _\text {B}$ ($-2.226$ $\mu _\text {B}$) in the second layer. The average of the moments of two Cr atoms, either in the first or second (or both) atomic layer(s) is $-3.114$ $\mu _\text {B}$ ($-3.132$ $\mu _\text {B}$) in the first layer and $-1.840$ $\mu _\text {B}$ ($-1.957$ $\mu _\text {B}$) in the second layer. The obtained magnetic moments are in line with the moments calculated for random substitutional Fe–Cr alloys using the coherent potential approximation^8^.

Next we consider the magnetic moments when oxygen is adsorbed in the bridge, hollow or on-top positions on the surface. Because we have calculated a large number of different Cr configurations, we present here only the moments at sites 1 (first layer) and 7 (second layer) and take an average over all calculated configurations with one Cr in the first or second layer (Table 3). The absolute value of the magnetic moments of first-layer Fe and Cr is reduced by the adsorbed O in all three adsorption sites. This reduction for Cr is much larger than for Fe. The effect of O on the moments in the second layer is generally smaller than in the first layer and both decrease and increase in the absolute value of the moment is obtained. Increasing the Cr content in Fe–Cr from the dilute limit to 9 at% changes the magnetic moments by less than 1%, except for the moment of Fe at site 1 ($-\,21$%) and the moment of Cr at site 7 ($-\,4$ %) when oxygen is adsorbed in the on-top position. The magnetic moment of oxygen is highest for the bridge adsorption (0.164 $\mu _\text {B}$ with the pure Fe surface) and lowest for the on-top adsorption (0.096 $\mu _\text {B}$ with two Cr atoms in the surface).

**Table 3:** Magnetic moments at atomic sites 1 and 7 (see Fig. 1) in the dilute-limit Fe–Cr alloy with one Cr atom in the first or second surface layer.

Adsorption site of O	Site 1				Site 7			
	$m_\text {Fe}$ ($\mu _\text {B}$)		$m_\text {Cr}$ ($\mu _\text {B}$)		$m_\text {Fe}$ ($\mu _\text {B}$)		$m_\text {Cr}$ ($\mu _\text {B}$)	
	With O	$\Delta m_\text {Fe}$	with O	$\Delta m_\text {Cr}$	with O	$\Delta m_\text {Fe}$	with O	$\Delta m_\text {Cr}$
br	2.790	$-\,0.078$	$-\,2.378$	0.755	2.351	0.108	$-\,2.297$	$-\,0.313$
ho	2.853	$-\,0.015$	$-\,3.041$	0.092	2.409	0.166	$-\,1.886$	0.098
ot	1.955	$-\,0.910$	$-\,1.734$	1.400	2.220	$-\,0.023$	$-\,1.921$	0.063

The Fe moments are averaged over Cr configurations with Cr in the nearest- or next-nearest-neighbour position to Fe. The magnetic moments (*m*) of Fe and Cr are shown with O adsorbed at either the bridge (‘br’), hollow (‘ho’) or on-top (‘ot’) site. The effect of the adsorbed O is measured by the difference $\Delta m_\text {X} = m_\text {X}(\text {with O}) - m_\text {X}(\text {without O})$, where $\text {X} = \text {Fe}$ or Cr. The magnetic moments without adsorbed O are $m_\text {Fe} = 2.868~\mu _\text {B}$ and $m_\text {Cr} = -\,3.133~\mu _\text {B}$ at site 1 and $m_\text {Fe} = 2.243~\mu _\text {B}$ and $m_\text {Cr} = -\,1.984~\mu _\text {B}$ at site 7.

## Discussion and summary

To gain atomic-level understanding of why oxygen bonding is stronger for some of the Cr-containing Fe surfaces than for the corresponding pure Fe surface, let us analyse the intricate interaction between iron, chromium and oxygen more closely. Previously Hu et al.^60^ reported that Cr in a Fe(110) surface changes the charge of neighbouring Fe atoms, thereby increasing their electron donor capabilities and prompting the adsorption of positive $\hbox {H}^{+}$ ions. In the case of oxygen on the (100) surface, we found the effect of Cr to be twofold. A Cr atom in the nearest-neighbour position to the Fe atom under the oxygen renders that Fe atom more attractive to oxygen regardless of the adsorption site. For the on-top adsorption, a Cr in any other neighbour position also renders the Fe atom (under oxygen) more attractive to oxygen except in the dilute alloy where a Cr at site 5 makes Fe less attractive to oxygen. For bridge and hollow adsorptions in both alloys, a Cr in a beyond-the-nearest-neighbour position renders Fe less attractive to oxygen. This means that a Cr atom within the two topmost surface layers produces an effective ‘driving force’ to escort a diffusing oxygen atom closer to the Cr atom. The energy difference that gives rise to this ‘driving force’ is, depending on the atomic configuration, about 0.06–0.38 eV, measured in terms of the oxygen adsorption energy. Our data sheds light on the issue of whether this Cr-induced change in the bonding between an oxygen atom and the surface is directly related to the changes in the atomic charges or not.

**Figure 7:**
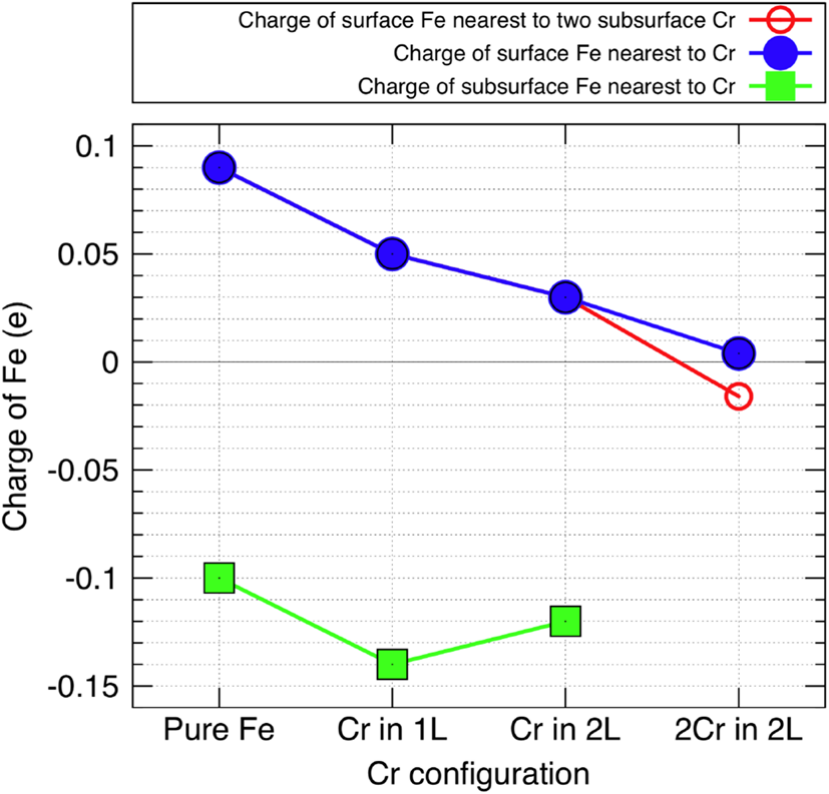
The charge of an Fe atom versus Cr configurations (with the neutral atom, of charge 0 *e*, as the reference level). The axis labels ‘Pure Fe’, ‘Cr in 1L’, ‘Cr in 2L’ and ‘2Cr in 2L’ refer to pure Fe surface, one Cr atom in the surface layer, one Cr atom in the subsurface layer and two Cr atoms in the subsurface layer at sites 8 and 9 (Fig. 1). The green curve with square markers shows the charge of the subsurface Fe atom nearest to the Cr atom. The blue curve with filled circles gives the charge of the surface Fe atom nearest to the Cr atom. For the ‘2Cr in 2L’ case, the red branch (open circle) shows the charge of the Fe atom at site 5, and the blue branch (filled circle) shows the charge of an Fe atom at sites 2 and 4.

Let us disregard the oxygen for a moment and consider oxygen-free Fe–Cr surfaces from the Bader-charge perspective. In a clean Fe surface, with no Cr, the Fe atoms in the surface and subsurface layers have electric charges of 0.09 *e* and - 0.10 *e*, respectively. But what happens to these charges when Cr is introduced to the surface? Let us analyze three different Cr configurations: (i) a single Cr atom in the surface layer; (ii) a single Cr atom in the subsurface layer; and (iii) two Cr atoms in the subsurface layer (positions 8 and 9 in Fig. 1). In case (i), the single Cr atom in the surface layer changes the charges of neighbouring surface-layer Fe atoms to 0.05 *e* and the charges of the nearest subsurface-layer Fe atoms to − 0.14 *e*. In other words, their Bader charges decrease by 0.04 *e* compared to the pure Fe surface case, indicating a net gain of electrons. In case (ii), where the single Cr atom is in the subsurface layer, the nearest Fe atoms in the surface layer have a charge of 0.03 *e*, and the nearest Fe atoms in the subsurface layer have a charge of − 0.12 *e*; therefore also in this case the Bader charges have decreased relative to the pure-Fe case. In case (iii), with two Cr atoms at the subsurface sites 8 and 9, the Fe atoms in the surface layer again acquire more electrons: Fe at site 5 between the two Cr atoms has a charge of − 0.02 *e*, and the Fe atoms at sites 2 and 4 have a charge of 0.00 *e*. The Cr-induced changes in the charges of Fe atoms are summarised in Fig. 7. Comparing these trends in the electronic charge transfer from Cr to Fe with our results for the oxygen adsorption shows that the extra electrons acquired by the Fe atoms from a nearby Cr atom are not generally available for forming stronger bonds between the iron and oxygen atoms. It would also be instructive to use other methods in addition to the Bader method to relate the changes in the atomic charges to observable physical quantities. For example, the Helmholtz method would provide a way to study surface polarization and the work function^55,62,63^.

The hollow, bridge and on-top adsorption sites for an oxygen atom were studied. The most favourable adsorption site in both investigated alloys was found to be the hollow site. Eleven different Cr configurations were studied for the hollow-site oxygen adsorption. For both alloys, the maximum variation among these 11 adsorption energies is about 0.6 eV. Among all the investigated adsorption sites and Cr configurations, the maximum variation in the oxygen adsorption energy is about 2.0 eV for the dilute alloy and 2.4 eV for the Fe_0.91_Cr_0.09_ alloy. The variation of the oxygen adsorption energy between different Cr configurations is generally larger among cases with two Cr atoms than among cases with one Cr atom. The adsorption energies of an oxygen atom on the Fe–Cr(100) surface, when analysed in order of magnitude, show clear steps and terraces (Fig. 3). That feature could be studied using the experimental techniques suitable for investigating energetics of adsorption^64^.

Insertion of different Cr-atom configurations into the two topmost atomic layers of a pure Fe surface can either increase or decrease the oxygen adsorption energies: Cr under an oxygen atom makes the oxygen bonding stronger and Cr farther away from the adsorption site makes the oxygen bonding weaker. This two-way effect is further enhanced when there are two Cr atoms in the surface layers. This demonstrates the general effect of Cr on the Fe surface: Cr attracts oxygen more than Fe and, at the same time, makes beyond-nearest-neighbour Fe atoms less attractive to oxygen than they would be in a pure-Fe surface. This Cr effect is strongest for bridge adsorption and weakest for on-top adsorption.

At the bridge site of the bcc Fe(100) surface, the shape of the minimum of the oxygen potential energy surface (PES) is very shallow along the minimum-energy diffusion path towards the hollow site compared to the shape of the minimum of the oxygen PES at the hollow site^65^. Consequently, the adsorption-energy difference between bridge-site and hollow-site oxygen gives a good approximation for the diffusion barrier of an oxygen atom escaping from a hollow site. For the Cr-free surface this difference is 0.65 eV (0.894 eV according to Cao et al.^65^), and for the hollow site with a Cr atom beneath the oxygen the difference is 0.91 eV. The lowest difference, 0.27 eV, is for oxygen moving from a hollow site to a bridge site between two Cr atoms. The highest difference, 1.09 eV, occurs for an oxygen atom moving from the strongest-bonding hollow-site configuration (Cr at sites 1 and 7) toward the bridge site between two iron atoms. All calculated differences are provided in Supplementary Information. All in all, the barrier analysis demonstrates that the oxygen affinity of chromium is higher than that of iron and, therefore, the surface diffusion of an oxygen atom on the bcc Fe–Cr(100) surface tends to be biased towards Cr atoms.

In summary, we have carried out ab initio density functional calculations to investigate the adsorption of atomic oxygen for two different Fe–Cr alloy compositions, namely, the dilute Fe–Cr alloy with the lattice constant of pure Fe and the Fe_0.91_Cr_0.09_ composition. Up to two chromium atoms were inserted in the two topmost surface layers. The two different investigated alloys were found to have the same order of preference for adsorption sites, hollow > bridge > on-top (from most to least favoured); the distances between the oxygen atom and the nearest metal atoms also turned out to be nearly identical for the two compositions. Although there were some difference in absolute adsorption energies, the relative adsorption energies were practically the same except in a few cases. The oxygen was found to prefer configurations that have a subsurface chromium atom right beneath the hollow adsorption site. A Cr atom was shown to reduce the oxygen affinity of Fe beyond the nearest neighbours of the Cr atom. This effect that the adsorption sites between the Cr sites become less favorable to oxygen, combined with the fact that the most favorable adsorption site of an oxygen atom is near to the Cr atom, leads to a biased oxygen diffusion probability towards Cr atoms and, thereby, an effective ‘pulling force’ that acts on the oxygen atoms towards the Cr atoms.

## Supplementary information

Supplementary Information.
