# Supplementary material for: Oxygen adsorption on (100) surfaces in Fe–Cr alloys
Source: Sci Rep. 2021 Mar 15;11:6046. doi: 10.1038/s41598-021-85243-0 (PMC7961028; doi:10.1038/s41598-021-85243-0)
Supplement: Supplementary file 1 — Supplementary Information. [file 41598_2021_85243_MOESM1_ESM.pdf]

Supplementary information for:

Oxygen adsorption on (100) surfaces in Fe-Cr alloys

Matti Ropo[1,2], Marko Punkkinen[1], Pekko Kuopanportti[3], Muhammad Yasir[1], Sari Granroth[1], Antti Kuronen[3], and Kalevi Kokko[1,*]

[1]Department of Physics and Astronomy, University of Turku, Turku, FI-20014, Finland
[2]Faculty of Engineering and Natural Sciences, FI-33014 Tampere University, Finland
[3]Department of Physics, University of Helsinki, P.O. Box 43, FI-00014 Helsinki, Finland
[*]kalevi.kokko@utu.fi

The total energies of the 45-atom unit cells (+ adsorbed oxygen) of dilute Fe-Cr and $Fe_{0.91}Cr_{0.09}$ alloys are shown in Supplementary Table S1.

The statistical probabilities (P) of the investigated atomic configurations of Cr in dilute Fe-Cr and $Fe_{0.91}Cr_{0.09}$ alloys at temperatures of 300 K, 700 K, 1100 K and 1500 K are estimated using the Maxwell–Boltzmann statistical distribution:

$$P_i(T) = \exp\left(\frac{-E_i}{kT}\right)\left[\sum_j \exp\left(\frac{-E_j}{kT}\right)\right]^{-1}, \tag{S1}$$

where i refers to a particular Cr configuration and the j sum is over the set of considered Cr configurations. To avoid biased energies between systems with different numbers of substituted Cr atoms, we consider the sets of one and two substitutional Cr atoms in the surface region as separate sets in the probability calculations. The obtained probabilities are shown in Supplementary Tables S2 and S3.

The 14 adsorption-energy differences ΔE_{P} of an oxygen atom on a (100) surface of an $Fe_{0.91}Cr_{0.09}$ alloy between a hollow and bridge adsorption sites have been determined using our calculated adsorption energies E_{ad}:

$$\Delta E_{\mathrm{P}} = E_{\mathrm{ad}}^{\mathrm{bridge}} - E_{\mathrm{ad}}^{\mathrm{hollow}}. \tag{S2}$$

The adsorption-energy differences and the corresponding atomic configurations are shown in Supplementary Figure S1.

The magnetic moments of Fe and Cr atoms in the surface layer and in the second layer, as well as the magnetic moments of the adsorbed O atom, are shown in Supplementary Tables S4 –S7. The average magnetic moments of Fe and Cr in the second layer are close to the values they have in the bulk Fe-Cr alloy of the same Cr concentration. In the surface layer, the absolute values of the magnetic moments of both elements are enhanced considerably. The magnetic moment of the oxygen atom varies from 0.053 μ_{B} to 0.164 μ_{B} depending on the adsorption site and the Cr configuration near the adsorption site.

Cr Pos	O pos	dilute Fe-Cr E (eV)	$Fe_{0.91}Cr_{0.09}$ E (eV)
-	clean	-386.702	-386.918
1	clean	-387.217	-387.382
7	clean	-386.839	-386.987
1-2	clean	-387.538	-387.664
1-5	clean	-387.620	-387.753
1-7	clean	-387.169	-387.261
1-8	clean	-387.299	-387.346
4-9	clean		-387.274
7-8	clean	-386.847	-386.859
7-10	clean	-386.971	-386.974
8-9	clean		-386.968
-	br	-394.539	-394.761
-	ho	-395.194	-395.413
-	ot	-393.711	-393.446
1	br	-395.366	-395.526
2	br	-395.003	-395.180
6	br	-395.000	-395.181
7	br	-394.744	-394.847
8	br	-394.550	
9	br	-394.595	-394.827
1	ho	-395.826	-395.989
3	ho	-395.680	-395.862
7	ho	-395.655	-395.768
9	ho	-395.315	-395.440
1	ot	-395.047	-395.236
2	ot	-395.364	-394.098
5	ot	-394.173	-394.351
7	ot	-393.960	-394.089
8	ot	-393.871	-393.952
1-2	br	-395.725	-395.841
1-4	br	-395.985	-396.111
1-5	br	-395.805	-395.930
1-7	br	-395.375	-395.448
2-5	br	-395.352	
2-7	br	-395.050	
2-9	br	-395.023	-395.230
8-9	br	-394.683	-394.785
1-2	ho	-396.259	-396.388
1-5	ho	-396.428	-396.549
1-7	ho	-396.142	-396.214
1-8	ho	-395.871	-395.900
4-9	ho	-395.769	-395.866
7-8	ho	-395.650	-395.672
8-9	ho	$-395,366$	-395.361
1-2	ot	-395.449	-395.603
1-5	ot	-395.521	-395.679
1-7	ot	-395.003	-395.109
1-8	ot	-395.133	-395.177
4-8	ot	-395.449	-395.488
8-9	ot	-393.999	-393.998

Table S1. Energies E of the dilute Fe-Cr and $Fe_{0.91}Cr_{0.09}$ alloys for different Cr ('Cr Pos') and oxygen ('O Pos') configurations ('clean' means no oxygen; for other notations, see the main text).

Cr config.	E (eV)	P(300 K)	P(700 K)	P(1100 K)	P(1500 K)
1	−387.217178	1.000 (0.995)	0.998 (0.870)	0.982 (0.714)	0.949 (0.598)
7	−386.839355	0.000 (0.001)	0.002 (0.051)	0.018 (0.118)	0.051 (0.159)
1-2	−387.537803	0.041 (0.001)	0.204 (0.057)	0.288 (0.137)	0.321 (0.187)
1-5	−387.619773	0.959 (0.999)	0.792 (0.934)	0.682 (0.810)	0.604 (0.689)
1-7	−387.168528	0.000 (0.000)	0.000 (0.008)	0.006 (0.040)	0.019 (0.076)
1-8	−387.299199	0.000 (0.000)	0.004 (0.000)	0.023 (0.002)	0.051 (0.009)
7-10	−386.971269	0.000	0.000	0.001	0.004
7-8	−386.846598	0.000 (0.000)	0.000 (0.000)	0.000 (0.000)	0.002 (0.002)

Table S2. Probabilities P of different configurations of Cr atoms in the dilute Fe-Cr alloy. E is the energy of the 45-atom unit cell, and P is the relative probability of one Cr atom (first two rows) or of two Cr atoms (last six rows) occuring at the designated sites in the surface or subsurface atomic layers. The column labelled 'Cr config' shows the positions of the Cr atoms (see Fig. 1 in the main text). The probability is calculated using the Maxwell–Boltzmann probability distribution at four different temperatures. (The values in parentheses correspond to the case where oxygen is adsorbed at the hollow site over atom 7.)

Cr config.	E (eV)	P(300 K)	P(700 K)	P(1100 K)	P(1500 K)
1	−387.381584	1.000 (0.992)	0.999 (0.870)	0.985 (0.729)	0.955 (0.622)
7	−386.986916	0.000 (0.000)	0.001 (0.022)	0.015 (0.710)	0.045 (0.113)
1-2	−387.663727	0.031 (0.002)	0.186 (0.065)	0.276 (0.150)	0.315 (0.203)
1-5	−387.752725	0.969 (0.998)	0.812 (0.931)	0.705 (0.814)	0.625 (0.701)
1-7	−387.260734	0.000 (0.000)	0.000 (0.004)	0.004 (0.024)	0.014 (0.053)
1-8	−387.346225	0.000 (0.000)	0.001 (0.000)	0.010 (0.001)	0.027 (0.005)
4-9	−387.273902	0.000 (0.000)	0.000 (0.000)	0.005 (0.001)	0.015 (0.004)
7-10	−386.973683	0.000	0.000	0.000	0.002
7-8	−386.859256	0.000 (0.000)	0.000 (0.000)	0.000 (0.000)	0.001 (0.001)
8-9	−386.967593	0.000 (0.000)	0.000 (0.000)	0.000 (0.000)	0.001 (0.000)

Table S3. Probabilities P of different configurations of Cr atoms in the $Fe_{0.91}Cr_{0.09}$ alloy. E is the energy of the 45-atom unit cell, and P is the relative probability of one Cr atom (first two rows) or of two Cr atoms (last eight rows) being located at the designated sites in the surface or subsurface atomic layers. The first column shows the positions of the Cr atoms (see Fig. 1 in the main text). The probability is calculated using the Maxwell–Boltzmann probability distribution at four different temperatures. (The values in parentheses correspond to the case where oxygen is adsorbed at the hollow site over atom 7.)

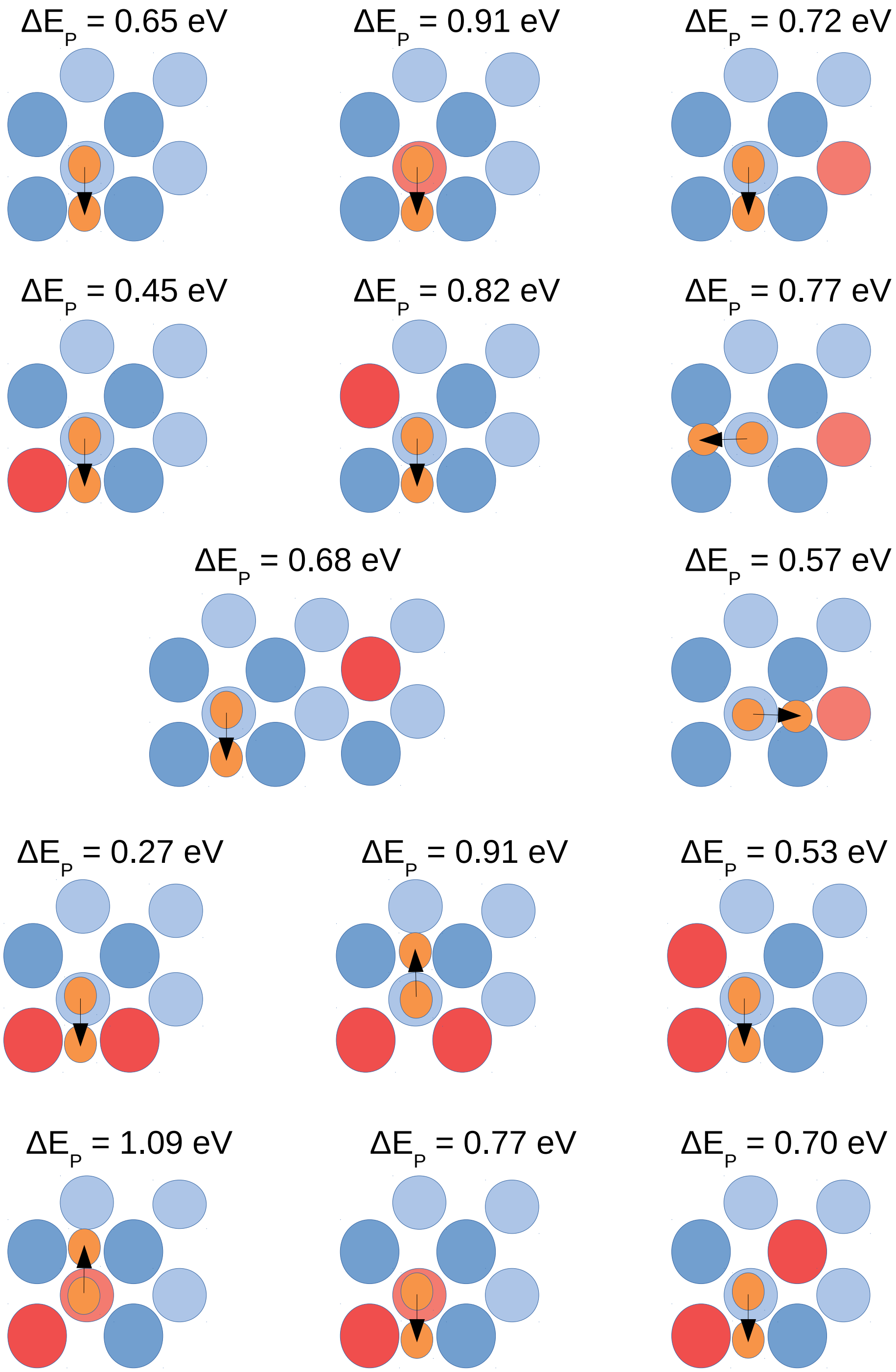

Figure S1. Adsorption-energy differences of an oxygen atom on the (100) surface of $Fe_{0.91}Cr_{0.09}$ between hollow and bridge adsorption sites. Blue, red and orange spheres are Fe, Cr and O atoms, respectively. Dark-coloured spheres are surface-layer atoms, and light-coloured ones are subsurface-layer atoms. The black arrow indicates the direction of the diffusion jump.

#Cr	SO	SCr	ST	$m\,(\mu_\mathrm{B})$	ST	$m\,(\mu_\mathrm{B})$	ST	$m\,(\mu_\mathrm{B})$	ST	$m\,(\mu_\mathrm{B})$	ST	$m\,(\mu_\mathrm{B})$	ST	$m\,(\mu_\mathrm{B})$
0	-	-	1Fe	2.971	2Fe	2.968	3Fe	2.968	4Fe	2.968	5Fe	2.966	6Fe	2.966
0	br	-	1Fe	2.860	2Fe	2.974	3Fe	2.974	4Fe	2.872	5Fe	2.972	6Fe	2.970
0	ho	-	1Fe	2.904	2Fe	2.912	3Fe	2.972	4Fe	2.912	5Fe	2.920	6Fe	2.968
0	ot	-	1Fe	1.788	2Fe	2.983	3Fe	2.983	4Fe	2.983	5Fe	2.964	6Fe	2.964
1	-	1	1Cr	-3.133	2Fe	2.884	3Fe	2.884	4Fe	2.884	5Fe	2.948	6Fe	2.948
1	-	7	1Fe	2.861	2Fe	2.854	3Fe	2.937	4Fe	2.854	5Fe	2.852	6Fe	2.933
1	br	2	1Fe	2.750	2Cr	-3.126	3Fe	2.887	4Fe	2.793	5Fe	2.881	6Fe	2.877
1	br	1	1Cr	-2.378	2Fe	2.888	3Fe	2.888	4Fe	2.891	5Fe	2.944	6Fe	2.951
1	br	8	1Fe	2.789	2Fe	2.863	3Fe	2.864	4Fe	2.808	5Fe	2.855	6Fe	2.952
1	br	7	1Fe	2.789	2Fe	2.888	3Fe	2.943	4Fe	2.786	5Fe	2.886	6Fe	2.944
1	br	6	1Fe	2.800	2Fe	2.875	3Fe	2.955	4Fe	2.798	5Fe	2.880	6Cr	-3.146
1	br	9	1Fe	2.823	2Fe	2.945	3Fe	2.987	4Fe	2.723	5Fe	2.854	6Fe	2.872
1	ho	1	1Cr	-3.041	2Fe	2.868	3Fe	2.890	4Fe	2.868	5Fe	2.931	6Fe	2.954
1	ho	3	1Fe	2.817	2Fe	2.825	3Cr	-3.128	4Fe	2.894	5Fe	2.906	6Fe	2.950
1	ho	7	1Fe	2.856	2Fe	2.856	3Fe	2.963	4Fe	2.856	5Fe	2.854	6Fe	2.960
1	ho	9	1Fe	2.886	2Fe	2.890	3Fe	2.975	4Fe	2.781	5Fe	2.786	6Fe	2.854
1	ot	2	1Fe	2.875	2Cr	-2.414	3Fe	2.899	4Fe	2.948	5Fe	2.882	6Fe	2.882
1	ot	1	1Cr	-1.734	2Fe	2.926	3Fe	2.926	4Fe	2.926	5Fe	2.966	6Fe	2.966
1	ot	8	1Fe	1.674	2Fe	2.880	3Fe	2.880	4Fe	2.958	5Fe	2.852	6Fe	2.941
1	ot	7	1Fe	1.676	2Fe	2.875	3Fe	2.966	4Fe	2.875	5Fe	2.874	6Fe	2.944
1	ot	5	1Fe	1.593	2Fe	2.894	3Fe	2.964	4Fe	2.894	5Cr	-3.144	6Fe	2.871
2	-	1-5	1Cr	-3.112	2Fe	2.771	3Fe	2.852	4Fe	2.771	5Cr	-3.100	6Fe	2.850
2	-	1-2	1Cr	-3.060	2Cr	-3.053	3Fe	2.767	4Fe	2.851	5Fe	2.848	6Fe	2.848
2	-	1-7	1Cr	-3.224	2Fe	2.767	3Fe	2.860	4Fe	2.767	5Fe	2.837	6Fe	2.919
2	-	1-8	1Cr	-3.137	2Fe	2.788	3Fe	2.788	4Fe	2.843	5Fe	2.835	6Fe	2.916
2	-	7-8	1Fe	2.844	2Fe	2.704	3Fe	2.823	4Fe	2.837	5Fe	2.695	6Fe	2.910
2	-	7-10	1Fe	2.859	2Fe	2.806	3Fe	2.885	4Fe	2.806	5Fe	2.720	6Fe	2.812
2	br	2-5	1Fe	2.727	2Cr	-3.069	3Fe	2.857	4Fe	2.730	5Cr	-3.063	6Fe	2.761
2	br	1-5	1Cr	-2.357	2Fe	2.792	3Fe	2.866	4Fe	2.807	5Cr	-3.115	6Fe	2.852
2	br	2-7	1Fe	2.713	2Cr	-3.247	3Fe	2.852	4Fe	2.753	5Fe	2.801	6Fe	2.856
2	br	1-2	1Cr	-2.281	2Cr	-3.102	3Fe	2.780	4Fe	2.863	5Fe	2.846	6Fe	2.861
2	br	1-7	1Cr	-2.557	2Fe	2.813	3Fe	2.859	4Fe	2.662	5Fe	2.849	6Fe	2.914
2	br	8-9	1Fe	2.757	2Fe	2.822	3Fe	2.857	4Fe	2.655	5Fe	2.764	6Fe	2.832
2	br	1-4	1Cr	-2.546	2Fe	2.859	3Fe	2.859	4Cr	-2.629	5Fe	2.853	6Fe	2.924
2	br	2-9	1Fe	2.737	2Cr	-3.132	3Fe	2.899	4Fe	2.679	5Fe	2.782	6Fe	2.805
2	ho	1-5	1Cr	-3.071	2Fe	2.804	3Fe	2.869	4Fe	2.804	5Cr	-3.048	6Fe	2.869
2	ho	1-2	1Cr	-3.010	2Cr	-2.990	3Fe	2.805	4Fe	2.856	5Fe	2.860	6Fe	2.864
2	ho	1-7	1Cr	-3.208	2Fe	2.806	3Fe	2.876	4Fe	2.806	5Fe	2.838	6Fe	2.941
2	ho	8-9	1Fe	2.858	2Fe	2.762	3Fe	2.863	4Fe	2.761	5Fe	2.663	6Fe	2.834
2	ho	1-8	1Cr	-3.040	2Fe	2.760	3Fe	2.807	4Fe	2.842	5Fe	2.807	6Fe	2.930
2	ho	7-8	1Fe	2.837	2Fe	2.828	3Fe	2.967	4Fe	2.694	5Fe	2.690	6Fe	2.865
2	ho	4-9	1Fe	2.846	2Fe	2.911	3Fe	2.957	4Cr	-3.097	5Fe	2.703	6Fe	2.872
2	ot	1-5	1Cr	-1.731	2Fe	2.820	3Fe	2.900	4Fe	2.820	5Cr	-3.106	6Fe	2.869
2	ot	1-2	1Cr	-1.600	2Cr	-3.138	3Fe	2.812	4Fe	2.898	5Fe	2.867	6Fe	2.867
2	ot	1-7	1Cr	-1.555	2Fe	2.837	3Fe	2.913	4Fe	2.837	5Fe	2.857	6Fe	2.942
2	ot	8-9	1Fe	1.700	2Fe	2.863	3Fe	2.885	4Fe	2.869	5Fe	2.746	6Fe	2.794
2	ot	1-8	1Cr	-1.684	2Fe	2.818	3Fe	2.818	4Fe	2.887	5Fe	2.850	6Fe	2.934
2	ot	4-8	1Fe	2.847	2Fe	2.842	3Fe	2.842	4Cr	-2.403	5Fe	2.756	6Fe	2.925
2	ot	2-4	1Fe	2.692	2Cr	-2.875	3Fe	2.908	4Cr	-3.209	5Fe	2.803	6Fe	2.882

Table S4. Magnetic moments m of the surface-layer atoms of the dilute Fe-Cr alloy. The column headers are as follows: '#Cr', number of Cr atoms; 'SO', site of oxygen ('-' is none, 'br' is bridge, 'ho' is hollow and 'ot' is on-top); 'SCr', site of Cr; 'ST', site and type of an atom. The site numbering is explained in the main text.

#Cr	SO	SCr	ST	$m\,(\mu_B)$	ST	$m\,(\mu_B)$	ST	$m\,(\mu_B)$	ST	$m\,(\mu_B)$	T	$m\,(\mu_B)$
0	-	-	7Fe	2.348	8Fe	2.340	9Fe	2.340	10Fe	2.332		
0	br	-	7Fe	2.442	8Fe	2.357	9Fe	2.364	10Fe	2.331	O	0.164
0	ho	-	7Fe	2.447	8Fe	2.411	9Fe	2.411	10Fe	2.291	O	0.125
0	ot	-	7Fe	2.278	8Fe	2.350	9Fe	2.350	10Fe	2.327	O	0.107
1	-	1	7Fe	2.228	8Fe	2.300	9Fe	2.300	10Fe	2.338		
1	-	7	7Cr	−1.984	8Fe	2.250	9Fe	2.250	10Fe	2.340		
1	br	2	7Fe	2.330	8Fe	2.226	9Fe	2.308	10Fe	2.293	O	0.146
1	br	1	7Fe	2.304	8Fe	2.294	9Fe	2.325	10Fe	2.339	O	0.110
1	br	8	7Fe	2.356	8Cr	−2.087	9Fe	2.407	10Fe	2.278	O	0.162
1	br	7	7Cr	−2.297	8Fe	2.257	9Fe	2.298	10Fe	2.354	O	0.147
1	br	6	7Fe	2.382	8Fe	2.310	9Fe	2.223	10Fe	2.204	O	0.150
1	br	9	7Fe	2.385	8Fe	2.400	9Cr	−2.175	10Fe	2.278	O	0.159
1	ho	1	7Fe	2.411	8Fe	2.388	9Fe	2.388	10Fe	2.292	O	0.106
1	ho	3	7Fe	2.416	8Fe	2.309	9Fe	2.413	10Fe	2.258	O	0.120
1	ho	7	7Cr	−1.886	8Fe	2.338	9Fe	2.338	10Fe	2.297	O	0.064
1	ho	9	7Fe	2.401	8Fe	2.431	9C	−2.097	10Fe	2.200	O	0.124
1	ot	2	7Fe	2.309	8Fe	2.294	9Fe	2.296	10Fe	2.317	O	0.112
1	ot	1	7Fe	2.211	8Fe	2.304	9Fe	2.304	10Fe	2.343	O	0.092
1	ot	8	7Fe	2.191	8Cr	−2.014	9Fe	2.363	10Fe	2.251	O	0.104
1	ot	7	7Cr	−1.921	8Fe	2.275	9Fe	2.275	10Fe	2.346	O	0.134
1	ot	5	7Fe	2.168	8Fe	2.228	9Fe	2.228	10Fe	2.209	O	0.071
2	-	1-5	7Fe	2.126	8Fe	2.177	9Fe	2.177	10Fe	2.209		
2	-	1-2	7Fe	2.174	8Fe	2.178	9Fe	2.248	10Fe	2.285		
2	-	1-7	7Cr	−1.815	8Fe	2.250	9Fe	2.250	10Fe	2.346		
2	-	1-8	7Fe	2.197	8Cr	−1.888	9Fe	2.297	10Fe	2.250		
2	-	7-8	7Cr	−1.797	8Cr	−1.636	9Fe	2.262	10Fe	2.268		
2	-	7-10	7Cr	−2.017	8Fe	2.178	9Fe	2.178	10Cr	−1.889		
2	br	2-5	7Fe	2.297	8Fe	2.169	9Fe	2.223	10Fe	2.190	O	0.140
2	br	1-5	7Fe	2.239	8Fe	2.172	9Fe	2.223	10Fe	2.209	O	0.106
2	br	2-7	7Cr	−2.162	8Fe	2.130	9Fe	2.262	10Fe	2.333	O	0.150
2	br	1-2	7Fe	2.268	8Fe	2.186	9Fe	2.282	10Fe	2.304	O	0.092
2	br	1-7	7Cr	−2.030	8Fe	2.226	9Fe	2.252	10Fe	2.350	O	0.107
2	br	8-9	7Fe	2.286	8Cr	−2.088	9Cr	2.187	10Fe	2.205	O	0.155
2	br	1-4	7Fe	2.156	8Fe	2.249	9Fe	2.264	10Fe	2.312	O	0.083
2	br	2-9	7Fe	2.304	8Fe	2.263	9Cr	−2.095	10Fe	2.230	O	0.144
2	ho	1-5	7Fe	2.318	8Fe	2.283	9Fe	2.283	10Fe	2.254	O	0.078
2	ho	1-2	7Fe	2.362	8Fe	2.265	9Fe	2.358	10Fe	2.247	O	0.078
2	ho	1-7	7Cr	−1.920	8Fe	2.327	9Fe	2.327	10Fe	2.304	O	0.060
2	ho	8-9	7Fe	2.343	8Cr	−1.979	9Cr	−2.006	10Fe	2.115	O	0.121
2	ho	1-8	7Fe	2.374	8Cr	−2.000	9Fe	2.394	10Fe	2.210	O	0.104
2	ho	7-8	7Cr	−1.692	8Fe	2.348	9Cr	−1.798	10Fe	2.209	O	0.053
2	ho	4-9	7Fe	2.340	8Fe	2.411	9Cr	−1.913	10Fe	2.203	O	0.100
2	ot	1-5	7Fe	2.092	8Fe	2.184	9Fe	2.184	10Fe	2.220	O	0.088
2	ot	1-2	7Fe	2.149	8Fe	2.210	9Fe	2.252	10Fe	2.304	O	0.068
2	ot	1-7	7Cr	−1.686	8Fe	2.253	9Fe	2.253	10Fe	2.358	O	0.088
2	ot	8-9	7Fe	2.116	8Cr	−2.094	9Cr	−2.046	10Fe	2.171	O	0.115
2	ot	1-8	7Fe	2.176	8Cr	−1.935	9Fe	2.318	10Fe	2.253	O	0.088
2	ot	4-8	7Fe	2.274	8Cr	−1.882	9Fe	2.315	10Fe	2.238	O	0.115
2	ot	2-4	7Fe	2.297	8Fe	2.286	9Fe	2.263	10Fe	2.274	O	0.107

Table S5. Magnetic moments m of the surface-layer atoms of the dilute Fe-Cr alloy. The column headers are as follows: '#Cr', number of Cr atoms; 'SO', site of oxygen ('-' is none, 'br' is bridge, 'ho' is hollow and 'ot' is on-top); 'SCr', site of Cr; 'ST', site and type of an atom. The site numbering is explained in the main text. The last column shows the magnetic moment of the adsorbed oxygen.

#Cr	SO	SCr	ST	$m\,(\mu_B)$	ST	$m\,(\mu_B)$	ST	$m\,(\mu_B)$	ST	$m\,(\mu_B)$	ST	$m\,(\mu_B)$	ST	$m\,(\mu_B)$
0	-	-	1Fe	2.961	2Fe	2.959	3Fe	2.970	4Fe	2.957	5Fe	2.971	6Fe	2.958
0	br	-	1Fe	2.826	2Fe	2.951	3Fe	2.969	4Fe	2.860	5Fe	2.969	6Fe	2.960
0	ho	-	1Fe	2.904	2Fe	2.898	3Fe	2.972	4Fe	2.903	5Fe	2.916	6Fe	2.960
0	ot	-	1Fe	1.752	2Fe	2.996	3Fe	2.996	4Fe	2.996	5Fe	2.974	6Fe	2.974
1	-	1	1Cr	−3.149	2Fe	2.858	3Fe	2.877	4Fe	2.859	5Fe	2.950	6Fe	2.940
1	-	7	1Fe	2.840	2Fe	2.837	3Fe	2.930	4Fe	2.833	5Fe	2.846	6Fe	2.919
1	br	2	1Fe	2.740	2Cr	−3.136	3Fe	2.879	4Fe	2.802	5Fe	2.880	6Fe	2.863
1	br	1	1Cr	−2.360	2Fe	2.868	3Fe	2.886	4Fe	2.881	5Fe	2.947	6Fe	2.944
1	br	7	1Fe	2.782	2Fe	2.871	3Fe	2.940	4Fe	2.775	5Fe	2.875	6Fe	2.926
1	br	6	1Fe	2.788	2Fe	2.847	3Fe	2.939	4Fe	2.816	5Fe	2.871	6Cr	−3.148
1	br	9	1Fe	2.793	2Fe	2.919	3Fe	2.972	4Fe	2.726	5Fe	2.834	6Fe	2.861
1	ho	1	1Cr	−3.037	2Fe	2.857	3Fe	2.898	4Fe	2.862	5Fe	2.930	6Fe	2.943
1	ho	3	1Fe	2.819	2Fe	2.821	3Cr	−3.184	4Fe	2.888	5Fe	2.894	6Fe	2.932
1	ho		1Fe	2.860	2Fe	2.850	3Fe	2.963	4Fe	2.859	5Fe	2.863	6Fe	2.950
1	ho	9	1Fe	2.878	2Fe	2.869	3Fe	2.975	4Fe	2.771	5Fe	2.772	6Fe	2.852
1	ot	2	1Fe	1.330	2Cr	−3.162	3Fe	2.904	4Fe	2.976	5Fe	2.880	6Fe	2.880
1	ot	1	1Cr	−1.724	2Fe	2.905	3Fe	2.923	4Fe	2.906	5Fe	2.965	6Fe	2.956
1	ot	8	1Fe	1.784	2Fe	2.869	3Fe	2.891	4Fe	2.949	5Fe	2.841	6Fe	2.927
1	ot	7	1Fe	1.655	2Fe	2.862	3Fe	2.962	4Fe	2.857	5Fe	2.866	6Fe	2.930
1	ot	5	1Fe	1.700	2Fe	2.877	3Fe	2.961	4Fe	2.877	5Cr	−3.177	6Fe	2.862
2	-	1-5	1Cr	−3.122	2Fe	2.751	3Fe	2.844	4Fe	2.751	5Cr	−3.134	6Fe	2.838
2	-	1-2	1Cr	−3.079	2Cr	−3.074	3Fe	2.765	4Fe	2.831	5Fe	2.854	6Fe	2.841
2	-	1-7	1Cr	−3.192	2Fe	2.744	3Fe	2.857	4Fe	2.735	5Fe	2.840	6Fe	2.901
2	-	8-9	1Fe	2.871	2Fe	2.788	3Fe	2.849	4Fe	2.787	5Fe	2.738	6Fe	2.790
2	-	1-8	1Cr	−3.132	2Fe	2.767	3Fe	2.773	4Fe	2.827	5Fe	2.823	6Fe	2.907
2	-	7-8	1Fe	2.827	2Fe	2.682	3Fe	2.806	4Fe	2.822	5Fe	2.690	6Fe	2.903
2	-	1-4	1Cr	−3.095	2Fe	2.832	3Fe	2.846	4Cr	−3.135	5Fe	2.850	6Fe	2.903
2	-	7-10	1Fe	2.847	2Fe	2.792	3Fe	2.881	4Fe	2.797	5Fe	2.723	6Fe	2.791
2	-	4-9	1Fe	2.842	2Fe	2.890	3Fe	2.941	4Cr	−3.222	5Fe	2.750	6Fe	2.832
2	br	1-5	1Cr	−2.331	2Fe	2.777	3Fe	2.860	4Fe	2.789	5Cr	−3.143	6Fe	2.844
2	br	1-2	1Cr	−2.246	2Cr	−3.103	3Fe	2.787	4Fe	2.839	5Fe	2.855	6Fe	2.855
2	br	1-7	1Cr	−2.585	2Fe	2.798	3Fe	2.858	4Fe	2.649	5Fe	2.856	6Fe	2.900
2	br	8-9	1Fe	2.744	2Fe	2.797	3Fe	2.838	4Fe	2.656	5Fe	2.743	6Fe	2.825
2	br	1-4	1Cr	−2.563	2Fe	2.836	3Fe	2.853	4Cr	−2.665	5Fe	2.856	6Fe	2.912
2	br	2-9	1Fe	2.710	2Cr	−3.127	3Fe	2.882	4Fe	2.669	5Fe	2.769	6Fe	2.784
2	ho	1-5	1Cr	−3.072	2Fe	2.793	3Fe	2.866	4Fe	2.797	5Cr	−3.094	6Fe	2.859
2	ho	1-2	1Cr	−2.996	2Cr	−3.001	3Fe	2.811	4Fe	2.847	5Fe	2.860	6Fe	2.858
2	ho	1-7	1Cr	−3.213	2Fe	2.802	3Fe	2.881	4Fe	2.809	5Fe	2.832	6Fe	2.927
2	ho	8-9	1Fe	2.843	2Fe	2.733	3Fe	2.854	4Fe	2.751	5Fe	2.654	6Fe	2.831
2	ho	1-8	1Cr	−3.002	2Fe	2.740	3Fe	2.803	4Fe	2.832	5Fe	2.788	6Fe	2.918
2	ho	7-8	1Fe	2.831	2Fe	2.820	3Fe	2.961	4Fe	2.703	5Fe	2.702	6Fe	2.848
2	ho	4-9	1Fe	2.853	2Fe	2.888	3Fe	2.947	4Cr	−3.096	5Fe	2.710	6Fe	2.862
2	ot	1-5	1Cr	−1.731	2Fe	2.803	3Fe	2.894	4Fe	2.802	5Cr	−3.159	6Fe	2.861
2	ot	1-2	1Cr	−1.602	2Cr	−3.136	3Fe	2.817	4Fe	2.880	5Fe	2.871	6Fe	2.859
2	ot	1-7	1Cr	−1.533	2Fe	2.798	3Fe	2.911	4Fe	2.788	5Fe	2.851	6Fe	2.929
2	ot	8-9	1Fe	1.925	2Fe	2.860	3Fe	2.856	4Fe	2.914	5Fe	2.726	6Fe	2.787
2	ot	1-8	1Cr	−1.609	2Fe	2.807	3Fe	2.817	4Fe	2.870	5Fe	2.839	6Fe	2.927
2	ot	4-8	1Fe	2.796	2Fe	2.817	3Fe	2.824	4Cr	−2.473	5Fe	2.747	6Fe	2.914

Table S6. Magnetic moments m of the surface-layer atoms of the $Fe_{0.91}Cr_{0.09}$ alloy. The column headers are as follows: '#Cr', number of Cr atoms; 'SO', site of oxygen ('-' is none, 'br' is bridge, 'ho' is hollow and 'ot' is on-top); 'SCr', site of Cr; 'ST', site and type of an atom. The site numbering is explained in the main text.

#Cr	SO	SCr	ST	$m\,(\mu_B)$	ST	$m\,(\mu_B)$	ST	$m\,(\mu_B)$	ST	$m\,(\mu_B)$	T	$m\,(\mu_B)$
0	-	-	7Fe	2.397	8Fe	2.411	9Fe	2.384	10Fe	2.399		
0	br	-	7Fe	2.460	8Fe	2.406	9Fe	2.387	10Fe	2.385	O	0.163
0	ho	-	7Fe	2.470	8Fe	2.468	9Fe	2.434	10Fe	2.370	O	0.130
0	ot	-	7Fe	2.316	8Fe	2.383	9Fe	2.383	10Fe	2.354	O	0.098
1	-	1	7Fe	2.248	8Fe	2.354	9Fe	2.322	10Fe	2.397		
1	-	7	7Cr	−2.026	8Fe	2.310	9Fe	2.283	10Fe	2.407		
1	br	2	7Fe	2.366	8Fe	2.270	9Fe	2.327	10Fe	2.337	O	0.148
1	br	1	7Fe	2.321	8Fe	2.335	9Fe	2.358	10Fe	2.379	O	0.111
1	br	7	7Cr	−2.295	8Fe	2.299	9Fe	2.317	10Fe	2.404	O	0.143
1	br	6	7Fe	2.396	8Fe	2.355	9Fe	2.255	10Fe	2.254	O	0.153
1	br	9	7Fe	2.382	8Fe	2.422	9Cr	−2.077	10Fe	2.317	O	0.161
1	ho	1	7Fe	2.414	8Fe	2.437	9Fe	2.404	10Fe	2.353	O	0.109
1	ho	3	7Fe	2.431	8Fe	2.350	9Fe	2.427	10Fe	2.318	O	0.123
1	ho	7	7Cr	−1.879	8Fe	2.400	9Fe	2.366	10Fe	2.373	O	0.063
1	ho	9	7Fe	2.414	8Fe	2.470	9Cr	−2.036	10Fe	2.275	O	0.128
1	ot	2	7Fe	2.194	8Fe	2.249	9Fe	2.316	10Fe	2.308	O	0.038
1	ot	1	7Fe	2.238	8Fe	2.356	9Fe	2.327	10Fe	2.384	O	0.093
1	ot	8	7Fe	2.233	8Cr	−2.251	9Fe	2.389	10Fe	2.317	O	0.121
1	ot	7	7Cr	−1.852	8Fe	2.321	9Fe	2.293	10Fe	2.394	O	0.141
1	ot	5	7Fe	2.186	8Fe	2.274	9Fe	2.240	10Fe	2.249	O	0.088
2	-	1-5	7Fe	2.141	8Fe	2.222	9Fe	2.190	10Fe	2.254		
2	-	1-2	7Fe	2.206	8Fe	2.226	9Fe	2.261	10Fe	2.324		
2	-	1-7	7Cr	−1.757	8Fe	2.297	9Fe	2.275	10Fe	2.394		
2	-	8-9	7Fe	2.211	8Cr	−2.258	9Cr	−2.015	10Fe	2.226		
2	-	1-8	7Fe	2.206	8Cr	−2.110	9Fe	2.318	10Fe	2.309		
2	-	7-8	7Cr	−1.775	8Cr	−1.735	9Fe	2.292	10Fe	2.321		
2	-	1-4	7Fe	2.211	8Fe	2.292	9Fe	2.208	10Fe	2.342		
2	-	7-10	7Cr	−2.067	8Fe	2.242	9Fe	2.211	10Cr	−2.138		
2	-	4-9	7Fe	2.159	8Fe	2.375	9Cr	−1.762	10Fe	2.297		
2	br	1-5	7Fe	2.238	8Fe	2.216	9Fe	2.233	10Fe	2.240	O	0.107
2	br	1-2	7Fe	2.278	8Fe	2.223	9Fe	2.301	10Fe	2.340	O	0.091
2	br	1-7	7Cr	−2.049	8Fe	2.273	9Fe	2.282	10Fe	2.398	O	0.112
2	br	8-9	7Fe	2.295	8Cr	−2.206	9Cr	−2.096	10Fe	2.247	O	0.158
2	br	1-4	7Fe	2.181	8Fe	2.297	9Fe	2.273	10Fe	2.367	O	0.087
2	br	2-9	7Fe	2.318	8Fe	2.284	9Cr	−1.993	10Fe	2.269	O	0.144
2	ho	1-5	7Fe	2.307	8Fe	2.327	9Fe	2.293	10Fe	2.297	O	0.080
2	ho	1-2	7Fe	2.352	8Fe	2.299	9Fe	2.370	10Fe	2.304	O	0.077
2	ho	1-7	7Cr	−1.904	8Fe	2.375	9Fe	2.345	10Fe	2.368	O	0.059
2	ho	8-9	7Fe	2.366	8Cr	−2.293	9Cr	−2.097	10Fe	2.181	O	0.123
2	ho	1-8	7Fe	2.384	8Cr	−2.158	9Fe	2.407	10Fe	2.270	O	0.107
2	ho	7-8	7Cr	−1.632	8Fe	2.397	9Cr	−1.731	10Fe	2.272	O	0.053
2	ho	4-9	7Fe	2.370	8Fe	2.453	9Cr	−1.834	10Fe	2.255	O	0.102
2	ot	1-5	7Fe	2.113	8Fe	2.228	9Fe	2.197	10Fe	2.260	O	0.089
2	ot	1-2	7Fe	2.168	8Fe	2.252	9Fe	2.267	10Fe	2.342	O	0.069
2	ot	1-7	7Cr	−1.558	8Fe	2.305	9Fe	2.284	10Fe	2.393	O	0.089
2	ot	8-9	7Fe	2.161	8Cr	−2.251	9Cr	−2.078	10Fe	2.232	O	0.139
2	ot	1-8	7Fe	2.187	8Cr	−1.939	9Fe	2.331	10Fe	2.312	O	0.084
2	ot	4-8	7Fe	2.289	8Cr	−1.977	9Fe	2.335	10Fe	2.295	O	0.118

Table S7. Magnetic moments m of the surface-layer atoms of the $Fe_{0.91}Cr_{0.09}$ alloy. The column headers are as follows: '#Cr', number of Cr atoms; 'SO', site of oxygen ('-' is none, 'br' is bridge, 'ho' is hollow and 'ot' is on-top); 'SCr', site of Cr; 'ST', site and type of an atom. The site numbering is explained in the main text. The last column shows the magnetic moment of the adsorbed oxygen.
